# Using mouse cursor tracking to investigate online cognition: Preserving methodological ingenuity while moving toward reproducible science

**DOI:** 10.3758/s13423-020-01851-3

**Published:** 2020-12-14

**Authors:** Martin Schoemann, Denis O’Hora, Rick Dale, Stefan Scherbaum

**Affiliations:** 1 Department of Psychology, Technische Universität Dresden, Zellescher Weg, 17, 01069 Dresden, Germany; 2 Department of Management, Aarhus University, Aarhus, Denmark; 3 School of Psychology, National University of Ireland, Galway, Ireland; 4 Department of Communication, University of California, Los Angeles, Los Angeles, CA USA

**Keywords:** Mouse cursor tracking, Experimental design, Response dynamics, Process tracing

## Abstract

Mouse cursor tracking has become a prominent method for characterizing cognitive processes, used in a wide variety of domains of psychological science. Researchers have demonstrated considerable ingenuity in the application of the approach, but the methodology has not undergone systematic analysis to facilitate the development of best practices. Furthermore, recent research has demonstrated effects of experimental design features on a number of mousetracking outcomes. We conducted a systematic review of the mouse-tracking literature to survey the reporting and spread of mouse variables (Cursor speed, Sampling rate, Training), physical characteristics of the experiments (Stimulus position, Response box position) and response requirements (Start procedure, Response procedure, Response deadline). This survey reveals that there is room for improvement in reporting practices, especially of subtler design features that researchers may have assumed would not impact research results (e.g., Cursor speed). We provide recommendations for future best practices in mouse-tracking studies and consider how best to standardize the mouse-tracking literature without excessively constraining the methodological flexibility that is essential to the field.

**Supplementary Information:**

The online version contains supplementary material available at 10.3758/s13423-020-01851-3.

Cognitive processes take time. Response time is the dominant measure of this cognitive processing. Indeed, the earliest attempts by Wundt and Donders to denote separable cognitive processes and investigate their interrelationships were informed by this metric. However, response times provide but a single constraint on the cognitive operations that happen between the stimulus and the response. Just more than 15 years ago, researchers began to employ mouse cursor tracking to investigate whether ongoing action was influenced by concurrent cognition. Because action was relatively continuously tracked during cognition, researchers surmised that features of ongoing action (e.g., deflection toward alternative responses) might provide further constraints on candidate sets of cognitive operations. This early period of mouse-tracking research has been characterized by impressive methodological ingenuity and has generated novel insights in a variety of cognitive subdomains. A downside of such ingenuity is, however, a lack of agreed standards that may inhibit the accumulation of knowledge. The current paper reviews the methodological degrees of freedom in mouse-tracking experiments and illustrates a path toward standards for mouse-tracking paradigms. Such standards can help this innovative technique reach a more mature phase of research methodology and thereby enhance reproducibility.

## Mouse-tracking as a process-tracing method

Process-tracing methods have a long tradition in psychological science. In order to understand cognition, researchers have employed introspective self-report measures such as verbal protocols (e.g., Ericson & Simon, 1984), as well as more objective behavioral or psychophysiological measures, such as eye-tracking (e.g., Russo & Rosen, 1975) or neuroimaging (e.g., Figner et al., 2010; for a comprehensive overview, please see Schulte-Mecklenbeck, Johnson, et al., 2017). Functional magnetic resonance imaging (fMRI), for instance, was introduced more than 25 years ago, and has been widely used in numerous domains of cognitive science (Sutterer & Tranel, 2017). Over the course of time, fMRI has been scrutinized and critiqued repeatedly (Logothetis, 2008; Moran & Zaki, 2013; Poldrack, 2008; Vul, Harris, Winkielman, & Pashler, 2009) in order to establish agreed standards in the field of fMRI (e.g., A. M. Dale, 1999; Friston, Zarahn, Josephs, Henson, & Dale, 1999) with varying results (for an overview, see Bandettini, 2012).

In the past 15 years, mouse cursor tracking has been added to the arsenal of methods available and has already made important contributions in many domains of psychological science (for recent reviews, see Erb, 2018; Freeman, 2018; Stillman, Shen, & Ferguson, 2018). Like many methods in psychological science, mouse cursor tracking has encouraged methodological ingenuity in experimental design and analysis. For instance, there are differences in how mouse cursor tracking is implemented across research domains and even between research groups within the same domain. These differences reflect demands of specific paradigms or phenomena, but also somewhat idiosyncratic intuitions about how mouse-tracking data might be collected, analyzed, and interpreted (Faulkenberry & Rey, 2014; Fischer & Hartmann, 2014; Hehman, Stolier, & Freeman, 2015). Such methodological variation is a consequence of a developing approach without agreed standards and has been important in sampling the range of potential paradigms that might be employed. Nevertheless, recent analyses suggest the methodological specifics of mouse-tracking paradigms (i.e., design features) influence the strength of the relationships observed between experimental manipulations and mouse-tracking outcomes (Grage, Schoemann, Kieslich, & Scherbaum, 2019; Kieslich, Schoemann, Grage, Hepp, & Scherbaum, 2020; Scherbaum & Kieslich, 2018; Schoemann, Lüken, Grage, Kieslich, & Scherbaum, 2019). This discovery of fundamental design issues suggests that codifying new standards for mouse-tracking research will facilitate strong reproducible findings that can more easily be combined across studies while creating a foundation for new methodological ingenuity (Morey et al., 2016; Munafò et al., 2017; Nosek et al., 2015).

In order to approach such standards, this review addresses three key aspects. First, it provides a brief introduction to the basics of mouse cursor tracking (i.e., paradigm and reasoning) and its potential variations. Second, the review discusses and integrates recent analyses suggesting that such methodological variations influence the conclusions drawn from mouse-tracking experiments. Finally, it examines the degree to which those methodological variations commonly occur in mouse-tracking paradigms.

## Basic paradigm and reasoning

**Fig. 1:**
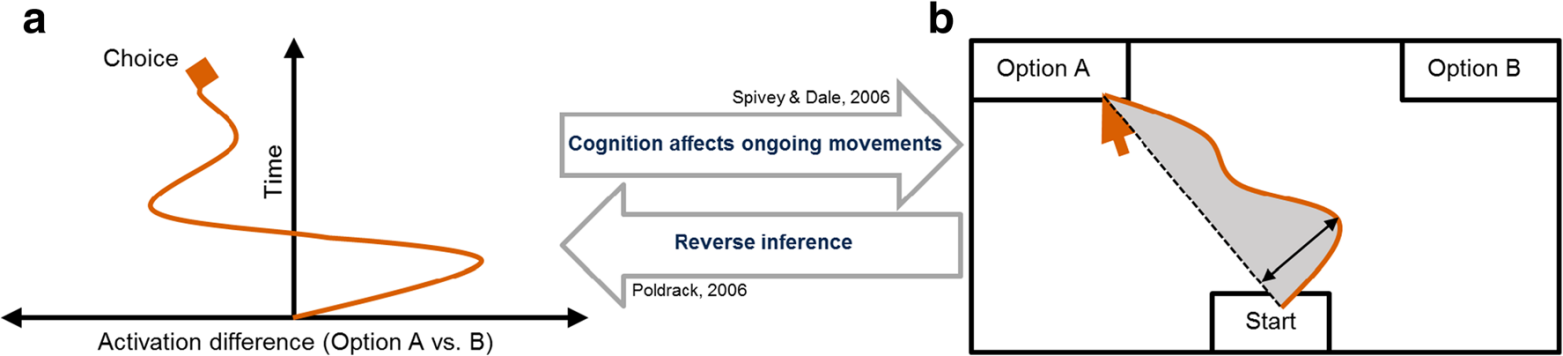
A simplified illustration of mouse cursor tracking as a process-tracing method. Cognitive processing (Panel **a**, on the left) is depicted as the activation difference between two options as a function of time. The corresponding continuous mouse cursor movement (Panel **b,** on the right) is depicted as the recorded cursor position (on the *x/y*-plane) in a basic mouse-tracking paradigm in which participants have to choose between two options, represented as response areas on a computer screen. Through a reverse inference (the lower arrow between the Panels **b** and **a**, from right to left), this cursor movement is taken as an indicator of the relative activation of the response options over the course of the decision-making process, assuming that the more an option is activated, the more the cursor trajectory deviates toward it (upper arrow, from left to right). Figure adapted from Wulff, Haslbeck, Kieslich, Henninger, and Schulte-Mecklenbeck (2018)

The canonical mouse-tracking paradigm involves a binary forced-choice task in which participants respond to an imperative stimulus by deciding between two options represented as buttons on a computer screen while their cursor movements are continuously recorded (see Fig. 1b for the basic setup and an exemplary cursor trajectory). These cursor movements are taken as an indicator of the relative activation of response options over the course of cognitive processing, assuming that the more an option is activated, the more the cursor trajectory deviates toward it (Spivey, Grosjean, & Knoblich, 2005). Thus, the degree of deflection (i.e., average deviation or maximum deviation from a notional straight line, e.g., O’Hora, Carey, Kervick, Crowley, & Dabrowski, 2016) is used as an indicator of the amount of activation or attraction to this option (see Fig. 1a for an exemplary trajectory of the options’ relative activation). More complex indicators have been used as well to infer properties of cognitive processes, such as the entropy of movements or the number of zero-crossings on the *x*-axis indicating conflict in the decision process (Calcagnì, Lombardi, & Sulpizio, 2017; Kieslich, Henninger, Wulff, Haslbeck, & Schulte-Mecklenbeck, 2019). In the end, this reasoning behind these indicators describes a reverse inference (Poldrack, 2006) that characterizes any behavioral or psychophysiological process-tracing method. For mouse-cursor tracking, the reverse inference is based on the assumption that cognitive processing affects ongoing motor activation/responses (e.g., hand movements) and hence cursor movements (Spivey & Dale, 2006), as depicted by two the unidirectional arrows in Fig. 1.

**Fig. 2:**
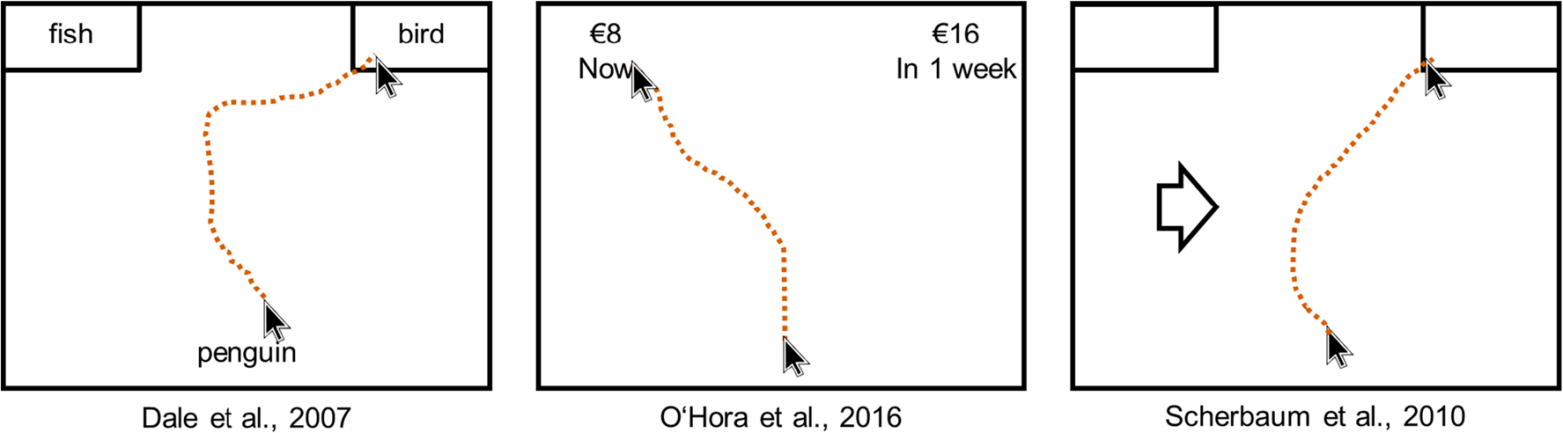
Sketches of the three exemplar studies

Since the first application in the area of language processing a decade and a half ago (Spivey et al., 2005), mouse cursor tracking has flourished in a broad range of psychological disciplines (for a reviews, see Erb, 2018; Freeman, 2018; Freeman, Dale, & Farmer, 2011; Lopez, Stillman, Heatherton, & Freeman, 2018; Stillman et al., 2018), not least because computer mice are very affordable and easy-to-use devices, with which most participants are well acquainted. This means responses in a computer-mouse task are of low technological complexity, and arguably very natural for participants. This simplicity is also mirrored by the fact that across many different areas of applications, the basic paradigm and reasoning has not changed substantially. However, paradigms differed with respect to seemingly small details of the mouse-tracking procedure. To provide a few examples of mouse-tracking applications and its unique procedure, we have selected three exemplar studies from our labs devoted to semantic processing, preferential choice, and action control. We present these three examples in the following paragraphs and highlight their methodological idiosyncrasies (Fig. 2).

The first study used mouse cursor tracking to investigate semantic processing (R. Dale, Kehoe, & Spivey, 2007), asking participants to classify exemplars (e.g., hawk or penguin) as belonging to one of two semantic categories (e.g., bird vs. fish). The mouse-tracking procedure in this experiment was described as follows (R. Dale et al., 2007, p. 17):

The participants were presented with two different animal category names, randomly assigned to one of the upper corners of a computer screen. After a 2,000-msec moment in which to view the category options, the text “Click Here” appeared in the bottom center of the screen. The participants were instructed to click first on that text and to wait for an animal word to appear in its place, then to click on the upper (left or right) category name that was appropriate for that animal. . . . Data . . . were collected by recording x- and y-coordinates of mouse movement trajectories. Due to occasional skipped samples, Psy- Scope’s sampling rate averaged approximately 42 Hz. As a result, each trial collected about 40–80 mouse position data points.

Hence, each trial started with the presentation of the response options located in the upper corners of the screen, and, with some delay, a start box located at the bottom center of the screen. After clicking the start box, the stimulus appeared with some delay in its place (i.e., bottom center of the screen), and participants had to click in the box of the respective response option to indicate their choice.

The second study used mouse cursor tracking to investigate intertemporal choice (O’Hora et al., 2016), asking participants to choose between a small reward that is immediately available and a large reward that is available after some delay. The mouse-tracking procedure in this experiment was described as follows (O’Hora et al., 2016, p. 14):

Two options were presented in each decision, and these were located in the top left and top right of the screen. Participants clicked a “Next” button at the button of the screen to begin a decision, and both options were presented simultaneously (the “Next button disappeared). Amount (e.g., €8) and delay (“Now”) information were presented simultaneously with amount above delay. Once participants clicked on one of the available options, the “Next” button at the bottom of the screen reappeared. . . . There was no overall time limit imposed on participants, but participants were prompted to complete a decision if they had not moved within 2 seconds.

Hence, each trial started with the presentation of a start box located at the bottom (may be center) of the screen. After clicking the start box, the stimuli appeared immediately in the upper corners of the screen, and participants had to click at the respective response option to indicate their choice. The cursor movement had to be started within a deadline of 2 s.

The third study used mouse cursor tracking to investigate action control in a Simon task (Scherbaum, Dshemuchadse, Fischer, & Goschke, 2010), asking participants to choose a left or right option depending on the direction of an arrow that was presented on the left versus right side. The mouse-tracking procedure in this experiment was described as follows (Scherbaum et al., 2010, p. 408):

Participants were asked to respond to the direction of a presented arrow by moving the mouse into the respective response box. Each trial consisted of three stages. In the first stage, participants had to click at a red box (11.55° in width) at the bottom of the screen within a deadline of 1.5 s. . . . Participants were required to start the mouse movement upwards within a deadline of 1.5 s. . . . Hence, only after moving at least 4 pixels in each of two consecutive time steps the third stage started with the appearance of the target stimulus. The trial ended after moving the cursor into one of the response boxes within a deadline of 2 s (see Fig. 1).

Hence, each trial started with the presentation of a start box located at the bottom center of the screen. After clicking the start box, participants had to move the cursor sufficiently (within a deadline of 1.5 s) to trigger stimulus presentation at the left or right center of the screen. Participants only had to hover into the respective response box to indicate their response with a deadline of 2 s.

In all three example experiments, participants had to click on a start button in the bottom center of the screen to start the trial (to align the starting position of the cursor across trials), but beyond this, the procedures differed substantially. First, they differed with respect to the start procedure and the response procedure—that is, the response requirements of the mouse-tracking task. In the first study, the stimulus appeared with some (fixed but not specified) delay after the click in the start box, and participants could indicate their response by clicking on one of the two response options; participants did not receive any specific instructions about how to move the cursor. Thus, the authors of the first study applied a *static start procedure* and a *click response procedure*. In the second study, the stimuli appeared immediately after the click in the start box, the response procedure did not differ, but participants were instructed to start cursor movement within 2 s introducing a movement initiation deadline. Thus, the authors of the second study applied a *deadline start procedure* and a click response procedure. In the third study, participants had to move the mouse upwards after the click in the start box for the stimulus to be displayed and could indicate their response by moving the cursor onto the corresponding button (no click was required); participants were instructed to start the cursor movement within 1.5 s and to finish responding within 2 s after stimulus presentation introducing a response deadline. Thus, the authors of the third study applied a *dynamic start procedure* and *hover response procedure*.

The three studies also differed with respect to the location of the elements within a trial, the placement of stimuli and response boxes—that is, the display characteristics of the experiment. In the first study, stimuli replaced the start box at the center bottom of the screen and the response boxes were located at the upper left and right corner of the screen. In the second study, the stimuli (i.e., rewards and delay for both options) were presented within the response boxes located in the upper left and right corner of the screen; the response boxes were dislodged from the screen’s corner and placed more toward the center thereby creating a small gap between the screen’s border and the response box. In the third study, stimuli were presented at the left and right center of the screen, and the response boxes were located at the upper left and right corner of the screen.

Taken together, these three studies vary considerably with regard to their mouse-tracking procedure, having implemented different response requirements (i.e., three unique start procedures and two unique response procedures), different characteristics of the experiment (i.e., two unique response box locations and three unique stimulus positions), as well as further hardware-related and software-related factors—mouse variables—not yet considered (e.g., the cursor speed settings, the sampling rates for the cursor movement). Some of those variations have already been discussed by the mouse-tracking community in recent years. Concerning the response requirements, Hehman et al. (2015) stressed the importance of instructing participants to initiate movements early by introducing a movement initiation deadline. Concerning the mouse variables, Fischer and Hartmann (2014) discussed the importance of the cursor speed^1^ settings and recommended the usage of slow cursor speed settings as well as clear reporting of those (regarding cursor speed, see also Huette, 2016).

## Review and synthesis of recent findings

Variations of the mouse-tracking procedure—design features of mouse cursor tracking—likely derived from idiosyncratic intuitions about potentially optimal procedures under a given task, rather than systematic investigations. Systematic investigation of these variations would serve empirically to answer the question of which specific design features offer which advantages and disadvantages and whether there might be an optimal mouse-tracking procedure for a particular phenomenon. Reverse-inference reasoning depends on a stable mapping between cognitive processing and observable measures (i.e., the mouse cursor movements; see Fig. 1; see also Schoemann, Schulte-Mecklenbeck, Renkewitz, & Scherbaum, 2019; Schulte-Mecklenbeck, Kühberger, Gagl, & Hutzler, 2017). Hence, the mouse-tracking setup should promote such a stable mapping rather than disturb it.

In this section, we summarize evidence that design features influence the integrity of this mapping. Four recent studies investigated the influence of the mouse-tracking setup on the consistency of the cursor movements within and across trials and the variety of movement types observed (Wulff, Haslbeck, Kieslich, Henninger, & Schulte-Mecklenbeck, 2019). First, consistency of movement within a trial refers to the extent to which the cursor moved continuously during the critical period when cognitive deliberation and response execution were both ongoing. To maximize the overlap between cognitive deliberation and response execution, cursor movements should ideally start at stimulus presentation and end with a final indication of the response, without any interruption in between. Interruptions of response execution (e.g., pausing) reduce the integrity of the cognition–movement mapping, since it is assumed that the underlying cognitive processes do not pause. Pauses in execution therefore induce misalignments between the ongoing processes. Second, consistency across trials refers to the extent that subjects move similarly from trial to trial. For example, some participants might stay at the starting point in difficult trials, but start to move immediately in easier trials. If so, cognitive processes would not influence early movement in difficult trials, which would undermine the integrity of the cognition–movement mapping that is inferred across trials. Finally, Wulff et al. (2019) identified a variety of trajectory prototypes that participants employed when making mouse cursor responses. These included straight line responses, curved responses, single change-of-mind responses, and double change-of-mind responses (see Fig. 4 for examples). The distribution of these prototypes can vary across conditions within a participant (change of mind responses are more likely in high conflict conditions) and across participants (some participants produce more straight line trajectories than others). These variations in distribution occlude or perturb the cognition–movement mapping than can be observed and inferred.

Scherbaum and Kieslich (2018) investigated the influence of the start procedure (static vs. dynamic) in a mouse-tracking version of a Simon task (Scherbaum et al., 2010). They found reliable Simon effects with comparable effect sizes in both start procedures, but revealed less consistent cursor movement within and across trials in the static compared with the dynamic start procedure. This decrease of the cursor movement consistency served as the first evidence that the static start procedure might perturb the mapping between cognitive processing and cursor movements. By doing so, these findings motivated further studies investigating the influence of the same or other design features.

Kieslich et al. (2020) investigated the influence of the start procedure, the response procedure, and the cursor speed setting in a semantic categorization task (R. Dale et al., 2007). Across all methodological setups, they replicated the postulated cognitive effect (category typicality effect), but revealed that the size of this effect was significantly influenced by the type of response and start procedure. Schoemann, Lüken, and colleagues (2019) investigated the influence of the start procedure, the response procedure, and the location of the stimuli in a mouse-tracking version of intertemporal choice task (Dshemuchadse, Scherbaum, & Goschke, 2013). They found that the variation of start procedure (i.e., static) perturbed the postulated cognitive effect; across the other methodological setups, they found no systematic variation of the cognitive effect. Grage et al. (2019) investigated the influence of the response procedure, the cursor speed setting, and the location of the response box in a mouse-tracking version of a Simon task (Scherbaum et al., 2010). Across all methodological setups, they found the postulated cognitive effect (Simon effect), but revealed that the size of this effect was significantly influenced by the type of response procedure.

**Fig. 3:**
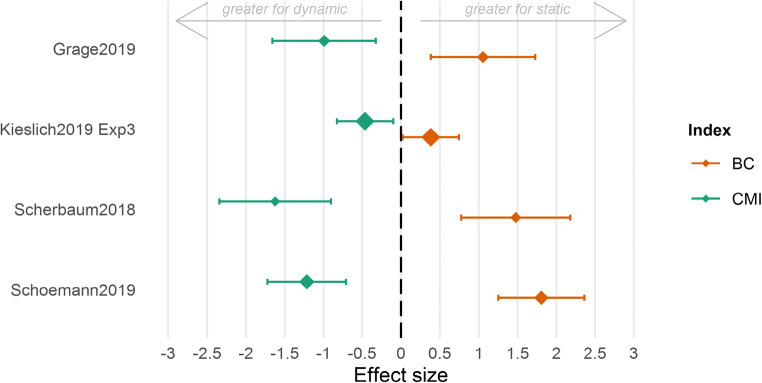
Effects of the start procedure (static vs. dynamic) on the consistency of the cursor movements. The consistency within trials is given by the continuous movement index (CMI, green); the consistency across trials is given by the bimodality coefficient (BC, orange). *Note*. The calculation of the effect size (g_s_) is based on two-tailed *t* tests comparing static versus dynamic conditions, hence a positive value indicates the effect being in favor of the static starting procedure, and vice versa. The size of the markers codes the weights of the studies in the accompanying meta-analysis (for detailed information, see Appendix 1)

**Fig. 4:**
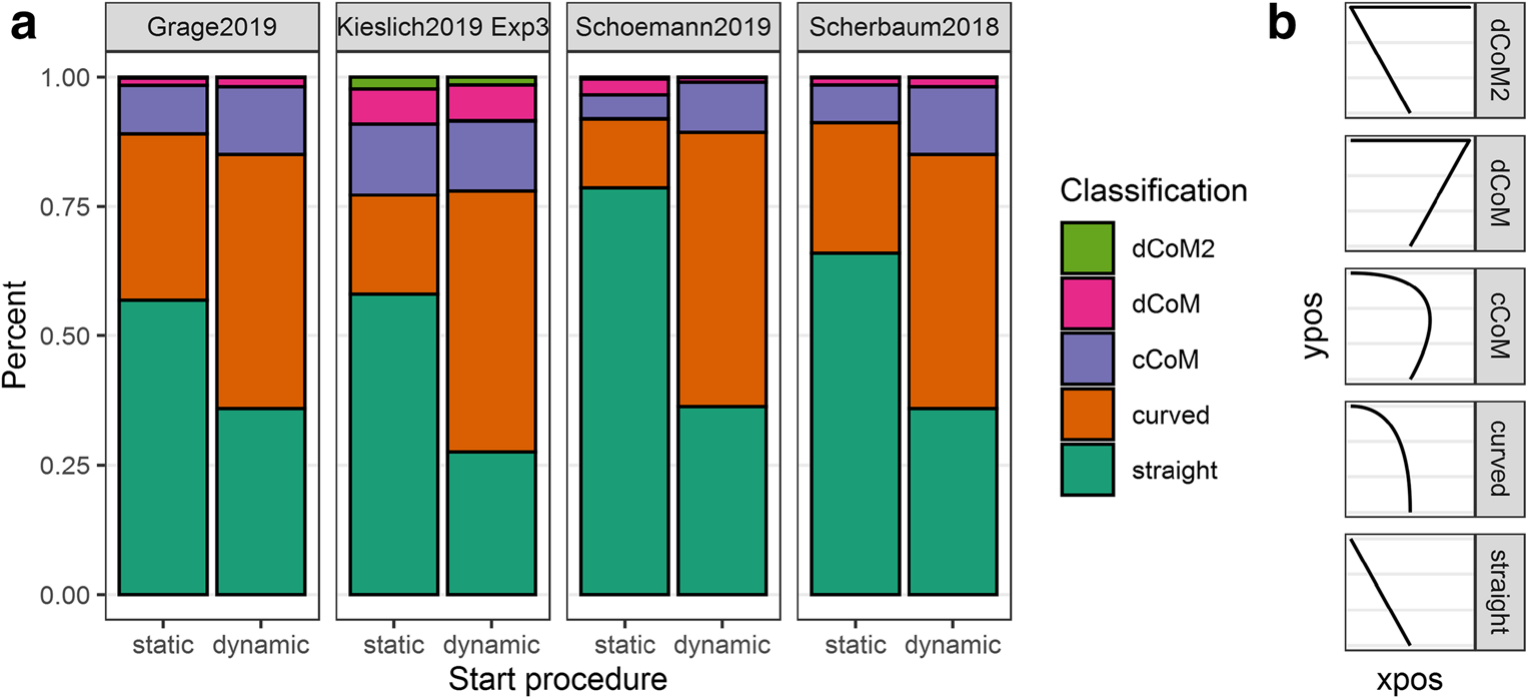
Distribution of trajectory types between start procedures (static vs. dynamic). Panel **a** depicts the cumulative proportion of the trajectory types separated by start procedure and study. Panel **b** depicts the five predefined trajectory prototypes which can be ordered (from bottom to top), for instance, with respect to the degree of response competition (i.e., competing activation of the options; see Fig. 1) they embody (Wulff et al., 2018)

The four studies described above compared performance using different start (static vs. dynamic) procedures. As an initial test of the effects of such procedures on the integrity of the cognition–movement mapping, we estimated movement consistency within and across trials (see Fig. 3) and the variety of trajectory prototypes observed under these conditions (see Fig. 4). Note that, in each case, we consider a different underlying cognitive process (for an overview of the cognitive processes as well as the effects between start conditions, please see Appendix 1), so the important comparisons are within each study. The consistency of cursor movements within trials was quantified via the continuous movement index (CMI; Scherbaum & Kieslich, 2018). This measure is calculated by correlating the observed *y*-axis positions of the cursor with a hypothetical constant and straight upward movement. The higher this correlation, the greater the cursor movement consistency within trials. This consistency of cursor movements across trials was quantified via the bimodality coefficient (BC) of each participant’s cursor movements (Freeman & Dale, 2013; Pfister, Schwarz, Janczyk, Dale, & Freeman, 2013). This coefficient can help to assess whether trials in an experiment induce a set of two or more distinct movement types, or “modes,” which would result in a bimodal distribution of, for instance, the deflection (see Fig. 1b). An experimental setup that causes variable response patterns (such as straight trajectories vs. more curved trajectories) would produce a higher bimodality coefficient—a more bimodal distribution of process measures. The lower this coefficient, the greater the consistency across trials.

Figure 3 unambiguously shows that the consistency of cursor movements within trials (as indexed by CMI) is higher for the dynamic start procedure compared with the static start procedure. The same result applies to the consistency of cursor movements across trials as the distribution of the average deviation of the cursor movement (as indexed by BC) is broader and potentially bimodally distributed in the static start procedure compared with the dynamic start procedure. This qualitative synthesis of the starting procedure’s effect is also supported quantitatively in a meta-analysis yielding a significant overall effect on both the cursor movement consistency within trials (CMI: *b* = −0.87, *SE* = 0.13, *z* = −6.74, *p* < .001, 95% CI [−1.12, −0.62]) and the consistency of cursor movements across trials (BC: *b* = 0.94, *SE* = 0.13, *z* = 7.13, *p* < .001, 95% CI [0.68, 1.19]). For more details about the studies and the meta-analysis, please see Appendix 1.

We also analyzed the distribution of movement (or trajectory) types (Wulff et al., 2019). First, we mapped the empirical movement trajectories of the four studies to a set of five predefined trajectory prototypes (see Fig. 4b) which can be ordered with regard to, for instance, the amount of conflict they mirror (Kieslich et al., 2019; Wulff et al., 2019), and then determined the effect of the start procedure on the distribution of the trajectory types via an ordinal mixed regression (e.g., Kieslich et al., 2020). Figure 4a consistently shows that a static start procedure increases the proportion of straight movement trajectories which is accompanied with a decreasing proportion of continuously curved trajectories. There is also a slight trend that a static start procedure increases the proportion of more discrete trajectories. This qualitative synthesis is also supported qualitatively in a meta-analysis yielding a significant overall effect of the start procedure (*b* = 1.22, SE = 0.13, *z* = 9.62, *p* < .001, 95% CI [0.97, 1.47]). For more details about the prototype mapping and the ordinal mixed regression in each study, and the meta-analysis, please see Appendix 1.

In sum, first systematic investigations on the influence of different mouse-tracking setups indicate two central findings. First, cognitive effects can vary substantially between different implementations of the start procedure. Second, those variations in cognitive effects were accompanied by variations in several characteristics of the cursor movement, such as the consistency of the cursor movements within and across trials as well as the distribution of movement types. These results indicate that it is worthwhile to investigate the relationship of design features and the mapping of the cognitive process to cursor movements, and that more methodological scrutiny might be necessary. However, these investigations so far were motivated and discussed based on merely hand-picked subsamples of different design features. To assess the importance of the obtained results for the past but also for future studies that will use mouse cursor tracking, it is important to arrive at a more representative picture of the current methodological ingenuity in the field.

## Systematic literature review

In order to survey the mouse-tracking studies in the literature, we followed a predefined systematic search protocol (available online at osf.io/nvcyx) which defined eligibility criteria, information sources, search strings, as well as the targeted mouse-tracking information to be extracted.

### Method

#### Eligibility criteria

We intended to include any study that applied a “classical” mouse-tracking paradigm as described above. Therefore, we defined this classical mouse-tracking paradigm as being characterized by apparatus, stimuli, and procedure in such a way that participants indicate their response to one (or more) imperative stimulus during each trial by moving a computer mouse toward one of several (usually two) response options. Furthermore, the location of the stimulus and the response options is arbitrarily constructed by the experimenter—that is, stimulus locations have no (contextual) meaning.^2^

Bearing this definition in mind, we selected experimental studies that (1) fulfilled our definition of a classical mouse-tracking paradigm; (2) were written in English, available in full-text format, published in peer-reviewed journals, and whose analyses were based on primary data; and (3) included only human participants.

#### Information sources

In order to maximize reproducibility and guarantee an unbiased search strategy, we limited our search to widely accepted electronic databases, and hence refrained from additionally searching Google Scholar, hand-picking references cited in highly relevant papers, or using references suggested by established researchers. The searched databases were Scopus, PubMed, and PsychInfo; all searches were conducted once from the database default start on November 22, 2018, and results were exported to .csv or .xml files.

#### Search strings

The search strings and concepts were: mouse tracking, mouse movements, mouse trajectories, cursor tracking. Appropriate truncation and wild cards were applied to these key-word concepts (e.g., trajector* or movem*). The search strategy was generated following Bramer and de Jonge’s (2015) guidelines on search standardization. This was adapted to each of the databases (when possible, the search command included a filter for human subjects; see Table 1).

**Table 1:** Search commands used for each of the databases

database	Search string strategy / command line
Scopus	TITLE-ABS-KEY(mouse?tracking OR(mouse PRE/0 tracking) OR cursor?tracking OR (cursor PRE/0 tracking) OR (mouse PRE/0 trajector*) OR (mouse PRE/0 movem*)) AND SRCTYPE(j) AND DOCTYPE (ar)
PubMed	(({mouse?tracking}[Title/Abstract] OR {mousetracking}[Title/Abstract] OR {cursor?tracking}[Title/Abstract] OR {cursortracking}[Title/Abstract] OR mouse trajector* [Title/Abstract] OR mouse movem*[Title/Abstract]) AND ("journal article"[Publication Type] OR systematic[sb]) AND "humans"[MeSH Terms])
PsychINFO	TI,AB,SU(mouse?tracking OR (mouse PRE/0 tracking) OR cursor?tracking OR (cursor PRE/0 tracking) OR (mouse PRE/0 trajector*) OR (mouse PRE/0 movem*)) AND PEER(yes)

#### Extracted data

We aimed to extract (and classify) the following details of the mouse-tracking procedure with the respective categories in parentheses (if applicable): start procedure (static, static with movement initiation deadline, dynamic), response deadline (true, false), response procedure (click, hover), response box location (upper corners, upper corners indented, circular), stimulus location (center, bottom center, upper center, full screen, in response box), cursor speed, sampling rate, training. We derived those details and the respective categories from initial informal screenings of the literature. The data extraction was solely conducted by the first author (M.S.), who did not follow a predetermined protocol. We did so, because during this more qualitative task, we intended to remain open to new, emerging categories. Due to one coder only, we are also not able to report any reliability measure with regard to category coding; however, the results are publicly available online for readers to review themselves (osf.io/nvcyx/).

### Results

**Fig. 5:**
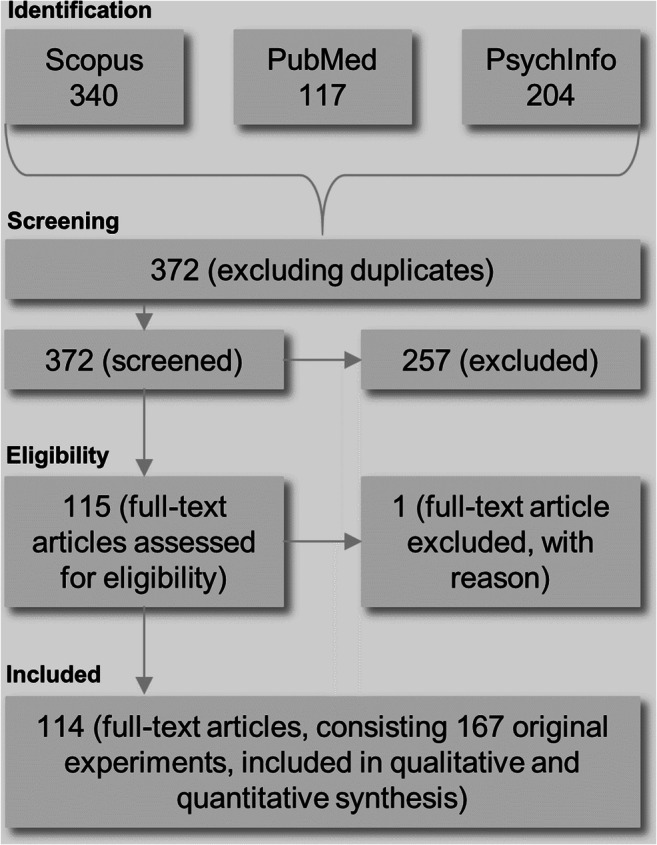
Flow diagram documenting the identification and processing of relevant studies throughout the systematic review process (Moher et al., 2015)

Our database search yielded 661 hits, of which 289 were identified as duplicates and excluded from subsequent screening. Hence, we screened 372 hits, of which 257 did not meet our eligibility criteria. From the remaining 115 articles that met our criteria, we had to exclude one^3^ article due to its research question. Consequently, we identified 114 articles consisting 167 original experiments that we included in our qualitative and quantitative synthesis (see Fig. 5).

**Fig. 6:**
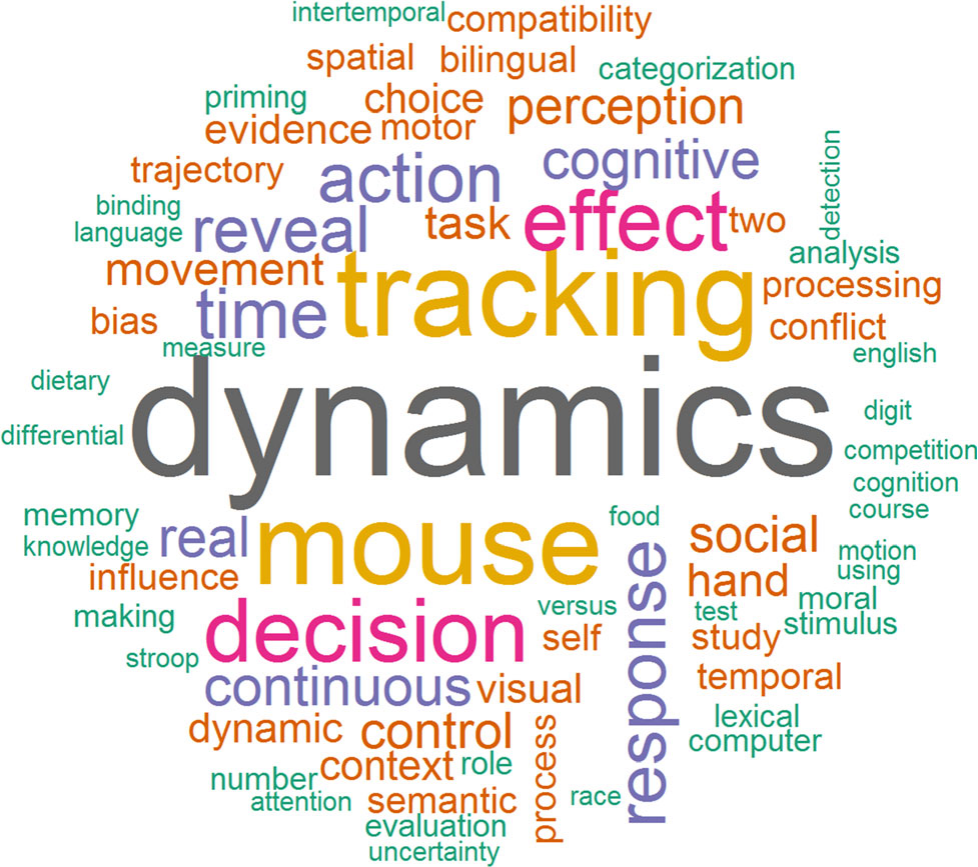
Word cloud visualizing the most frequent terms used in the titles of all identified full-text articles. *Note*. Font size and color represent the (relative) frequency; the word cloud was compiled using the *wordcloud* package (Fellows, 2018) in R (see analysis script online at osf.io/nvcyx/)

Indeed, the identified articles cover a broad range of psychological domains as argued by several recent reviews (Erb, 2018; Freeman, 2018; Freeman et al., 2011; Lopez et al., 2018; Stillman et al., 2018). In Fig. 6, we depicted a so-called word cloud of the terms that had been frequently utilized in the titles of all 114 identified full-text articles. Among those, the relatively frequent terms *decision*, *semantic*, *bilingual*, and *social* support the notion that mouse cursor tracking has exceptionally flourished in the domains of decision-making, linguistics, and social psychology. However, the most frequent terms *dynamics*, *mouse*, and *tracking* largely refer to the process-tracing method used—which is not surprising when a relatively new method is applied—as well as reflect the search strings used in our systematic search.

After the identification of the 167 original experiments, we extracted (and classified) the defined design features. However, before we can turn to the results of those classifications, we would like to emphasize an issue that we did not anticipate in advance, but which became very salient during data extraction—the extent of reporting.

#### Reporting

**Fig. 7:**
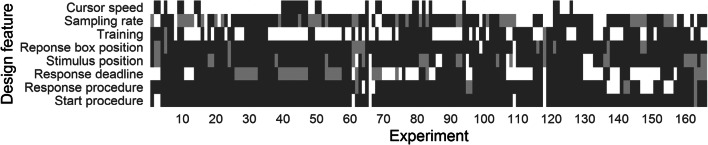
For each original experiment (on the *x*-axis) and information, we add a dark-gray rectangle if the information is reported in the respective text, a light-gray rectangle if the information is otherwise available (e.g., through inspecting figures or following references), and a white rectangle if the information is completely unavailable

For our systematic review, we wanted to extract and classify eight design features of the identified studies. Trivially, this is only possible if this information is provided. For an overview of the extent of reported design features, we coded the presence or absence of our set of required information. In Fig. 7, we added a dark-gray black rectangle given that the design feature was reported in the paper text. A light-gray rectangle was added, given that the design feature was otherwise available through, for instance, visual inspection of figures or following a given reference. A white rectangle was added whenever the design feature was completely missing and could not be inferred otherwise.

As indicated by Fig. 7, only 1.81% of the studies completely reported information on all design features, and for only 4.82% of the studies, the information on all design features is available due to explicit reporting or can be deduced via figures or references; on an individual level, cursor speed is the least reported feature (only 16.87% of studies; see Table 2). This indicates that reproducibility of mouse-tracking results may be difficult not *just* because of methodological ingenuity that is typical for a young field, but also because of an equally important practice: Extensive and detailed reporting without which reproducibility is not achievable in principle (Goodman, Fanelli, & Ioannidis, 2016; Munafò et al., 2017; Nosek et al., 2015; Wicherts et al., 2016). This lack of extensive reporting means that our following analysis will always be limited to the studies for which we could identify the respective design feature. We will hence report the sample size for each analysis to allow for a clear interpretation of the results.

**Table 2:** Relative frequency (in %) of the quality of reporting for each design feature separately

	Design feature	Reported	Deducible	None
Mouse variables	Cursor speed	16.87	0.00	83.13
	Sampling rate	60.84	22.86	16.27
	Training	53.01	1.20	45.78
Physical characteristics	Response box position	90.36	8.43	1.20
	Stimulus position	78.92	15.06	6.02
Response requirements	Response deadline	51.81	18.67	29.52
	Response procedure	87.35	3.61	9.04
	Start procedure	95.78	0.60	3.61

#### Response requirements

**Fig. 8:**
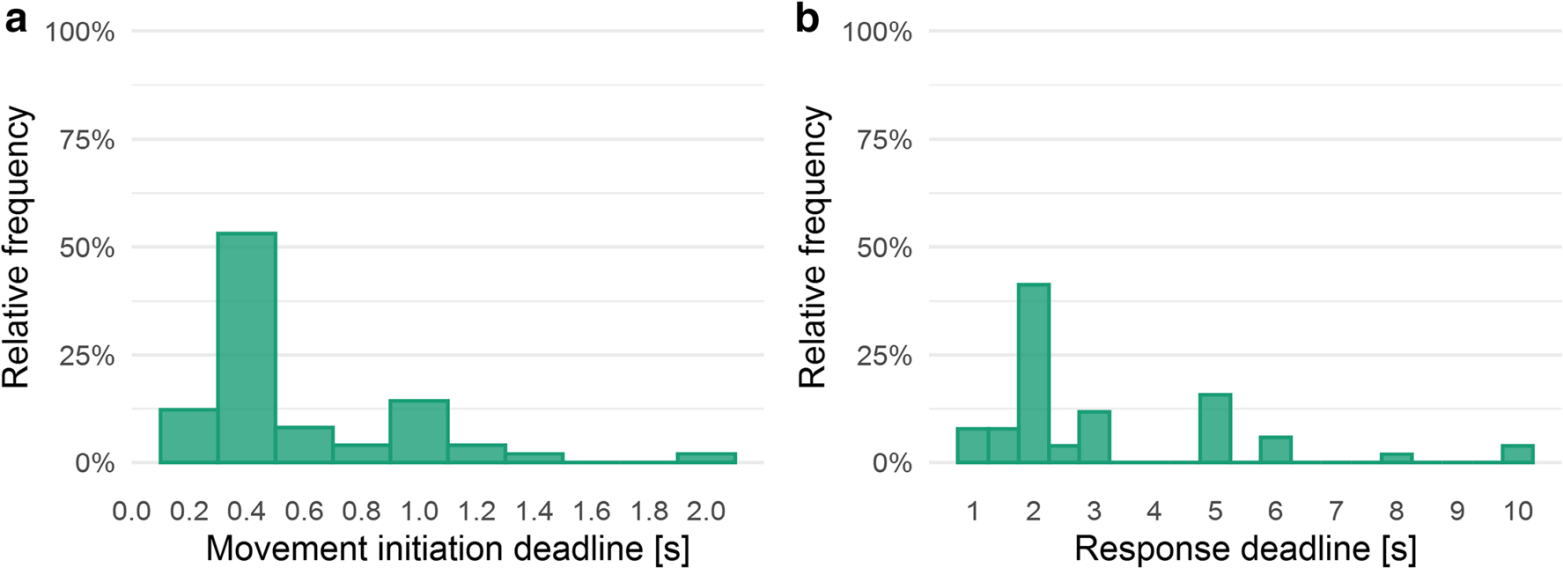
**a** Relative frequency (in percentage, *n* = 50) of the movement initiation deadline (bin width = 0.2). **b** Relative frequency (in percentage, *n* = 51) of the movement initiation deadline (bin width = 0.5)

Concerning the implemented start procedures, we distinguished between three types: *static*, *deadline*, and *dynamic*. In a static start procedure, the stimuli appear either immediately or with some delay after participants have clicked the start box, and no further instructions for the cursor movements are made. The deadline start procedure is implemented the same way as the static start procedure, though participants are instructed to initiate cursor movement early—that is, within a specified movement initiation deadline. In a dynamic start procedure, the appearance of the stimuli is connected to the upward cursor movement after participants have clicked the start box. In 160 studies, we found a preference for the static start procedure (*n* = 96, 60.00%), followed by deadline (*n* = 50, 31.25%), and dynamic (*n* = 13, 8.13%). Within deadline, the movement initiation deadline ranged from 250 ms to 2,000 ms (*M* = 624.5 ms, *SD* = 357.82 ms), revealing a high variance of this parameter across experiments (see Fig. 8a).

Concerning the implemented response procedures, we distinguished between three types: *click*, *hover*, and *deadline*. In a click response procedure, participants indicate their response/choice by clicking onto the respective response box. In a hover response procedure, participants indicate their response by moving the cursor onto the respective response box; the response is indicated as soon as the cursor enters the response box, no additional click is required. The deadline response procedure is implemented analogously to hover, though the response is not indicated as soon as the cursor enters the response box, but after a certain delay for which the cursor stayed in the respective area. In 153 studies, we found a preference for the click response procedure (*n* = 127, 83.01%), followed by hover (*n* = 25, 16.34%), and deadline (*n* = 1, 0.65%). We also extracted information about response deadlines. In 116 studies, 51 studies applied a response deadline ranging from 930 ms to 10,000 ms (*M* = 3,168 ms, *SD* = 2,122.25 ms; see Fig. 8b).

#### Physical characteristics

Concerning the implemented response box position, we distinguished between four types: *corner*, *medial*, *circular*, and *other*. A corner response box position defines that the response boxes are located directly in the top corners of the screen (see Fig. 1b). A medial response box position defines that the response boxes are dislodged from the screen’s corners and placed more toward the center thereby creating a small gap between the screen’s border and the response boxes. A circular response box position defines that the response boxes are also located below or next to instead of above the cursor’s starting position (usually the start box). The last category defines all response box positions that did not fall into the former three. In 164 studies we found a preference for the corner response box position (*n* = 104, 63.41%), followed by medial (*n* = 31, 18.90%), other (*n* = 17, 10.37%), and circular (*n* = 12, 7.32%).

Concerning the implemented stimulus position, we distinguished seven types: *center*, *lower center*, *upper center*, *in response box*, *full screen*, *other*, and *phono*. The former three types define that the stimulus is presented in the screen’s vertical center, or below, or above, respectively. The other types define that the stimulus is presented within the response boxes, all over the screen, somehow differently, or via audio. In 156 studies, we found a preference for a stimulus presentation in the screen’s center (*n* = 75, 48.08%), followed by a presentation below the screen’s center (*n* = 34, 21.79%), within the response boxes (*n* = 20, 12.82%), and an auditory presentation of the stimulus (*n* = 14, 8.97%). The remaining types have only rarely been used (*n* = 13, 8.33%).

#### Mouse variables

**Fig. 9:**
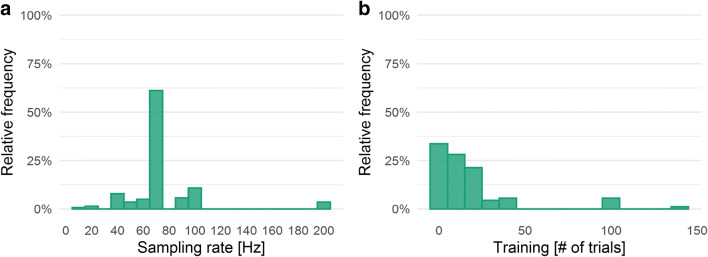
**a** Relative frequency (in percentage, *n* = 139) of the sampling rate (bin width = 10). **b** Relative frequency (in percentage, *n* = 89) of the number of training trials (bin width = 10)

The sampling rate defines the frequency of registering the cursor’s x and y coordinates per second (Hz). We collected 139 sampling rates ranging from 5 Hz to 200 Hz (*M* = 73.63 Hz, *SD* = 29.68 Hz), see Fig. 9a.

As training, we defined the number of trials in a paradigm that are used to familiarize participants with the respectively employed mouse-tracking procedure. In 89 studies, we found statements about training/practice trials whose quantity ranged from two to 144 trials (*M* = 19.44 trials, *SD* = 26.21 trials; see Fig. 9b). However, this distribution is most likely biased, since the number of training trials might have only been explicitly provided if training trials have been employed. So, one could assume that the studies in which no information on training trials have been given just did not include training in their paradigm. Against this interpretation stands the observation that some studies from our own labs did not report training though training trials have been employed.

Concerning cursor speed, we only collected 28 specifications which differed substantially in their quality. Many unspecific descriptions stated that the operating system’s default settings or changes relative to those settings were used (n = 11), sometimes in combination with the information that the operating system’s nonlinear cursor acceleration was enabled (*n* = 1) or disabled (*n* = 1). Few equally unspecific descriptions only stated that the operating system’s nonlinear cursor acceleration was disabled (*n* = 2), or stated that a not further specified setting of the MouseTracker software (Freeman & Ambady, 2010) was used (*n* = 2). Only 11 studies stated the hand-to-cursor movement ratio as recommended by (Fischer & Hartmann, 2014). Those ratios range from 0.3 cm per 100 px (*n* = 2), over 1.0 cm per 100 px (*n* = 8), to 1.25 cm per 100 px (*n* = 1). For those specifications, it is most likely that the operating system’s nonlinear cursor acceleration was disabled but this was only explicitly reported for eight experiments.

#### Typical protocol

So far, we have only evaluated frequencies on categories of individual design features. To obtain an even better understanding of the methodological ingenuity in the mouse-tracking research, we also investigated whether there is a typical protocol that uses the same implementations of several design features. Due to the identified lack of reporting or continuous data, we did so in consideration of the response requirements and the physical characteristics only, without taking specific values of the movement initiation time or response deadline into account. In 107 studies for which those design features were available (i.e., reported or deducible; see Fig. 7 and Table 2), we identified two typical protocols (*n* = 9 each). One protocol implemented a dynamic starting procedure with a response deadline, a hover response procedure with response boxes in the two upper corners of the screen, and a centered (visual) stimulus. The other protocol implemented a static starting procedure without a response deadline, a click response procedure with response boxes in the two upper corners of the screen, and a (visual) stimulus that positioned in the lower center of the screen. For the former, the typical response deadline might range from 2 s to 2.5 s. For both, the typical sampling rate might range from 70 Hz and 80 Hz, and the cursor speed might remain at the default setting of the respective operating system. The amount of training might range from zero to 10 trials, though it is reasonable to assume that the first typical protocol might demand for more training due to the response deadline and the dynamic start procedure for which participants must learn how to move the cursor in order to satisfy the procedural constraints of the respective paradigm. This learning aspect does not apply to the second typical protocol in which almost no constraints are given and participants use the cursor as in any other desktop application. Hence, one could also assume that the amount of training might serve as a proxy for a hidden design feature—namely, whether participants know that their cursor movements are recorded. The more emphasis is put on the mouse-tracking procedure, the more likely the participants might suspect the recording of their cursor movements. In fact, there are only a few studies explicitly reporting that participants were unaware of those recordings.

## General discussion

In the past 15 years, mouse cursor tracking has been applied in many domains of psychological science (Erb, 2018; Freeman, 2018; Song & Nakayama, 2009; Stillman et al., 2018). With our review, we briefly summarized the history of mouse-tracking research, highlighting its impressive methodological ingenuity. We summarized how past mouse cursor tracking has been conducted, indicating the degrees of freedom in setting up mouse-tracking experiments, and we synthesized recent evidence suggesting that such degrees of freedom should be carefully handled because the unique implementation of design features can affect experimental results. In the next section, we will discuss the implications of our findings for the past and the future of mouse-tracking research.

### Are design features important?

We reviewed and synthesized recent findings on design features in mouse cursor tracking (Grage et al., 2019; Kieslich et al., 2020; Scherbaum & Kieslich, 2018; Schoemann, Lüken, et al., 2019) that indicate a considerable influence of experimental design choices on cognitive effects as measured by mouse cursor tracking.

For instance, Kieslich et al. (2020) showed that across four variants of the start procedure the mouse-tracking effect of interest varied substantially, with the dynamic start procedure yielding the smallest, and the static start procedure with a movement initiation deadline yielding the largest effect. As a more extreme example, Schoemann, Lüken, and colleagues (2019) showed that—comparing the static and the dynamic start procedure—the effects of interest not only varied quantitatively but also qualitatively in such a way that a static start procedure led to an extinction of the effect.

Further converging results have been reported for different variants of the response procedure and different settings of the cursor speed. Thus, Kieslich et al. (2020) showed that the click response procedure yielded larger effects than the hover response procedure, and that a default, relatively fast cursor speed yielded larger effects than a reduced, relatively slow cursor speed. Grage et al. (2019) showed that the click response procedure yielded larger Simon effects as given by discrete and continuous mouse-tracking measures than the hover response procedure, and that a fast cursor speed yielded larger Simon effects than a slow cursor speed. Crucially, all those variations in the cognitive effects of interest were accompanied by variations in the consistency of the cursor movements.

Together these findings suggest that certain experimental design choices influence the validity of the cursor movement as a process measure for cognitive processing. In other words, design choices vary the amount of cognitive processing that can be detected in cursor movements.

For instance, a static starting procedure might inconsistently influence participants, because it leaves open different strategies for them to handle the task, thus complicating the relationship between cognition and cursor movements. Participants could choose either to execute cursor movements while processing the relevant information to solve the task or to finish processing first (while standing still with the cursor), and *then* move into the response box. In the latter case, the cursor movement might only reflect the response selection instead of the cognitive processes to solve the task. In contrast to this freedom in the static start procedure, a dynamic start procedure ensures that participants execute cursor movements while processing the relevant information to solve the task, and makes it more likely that cognitive processing will be reflected in cursor movements (Hehman et al., 2015). However, as possible downside of this procedure, the restricted freedom might cause any covariation with pure cognitive processing to be confounded with other processes that are needed to fulfill the procedural constraints of the task.

Similar intuitions can be expressed with regard to the response procedure. A click response procedure might enhance the coupling because participant can execute cursor movements early and freely without fearing erroneous responses by accidently reaching the potentially wrong response option. However, according to the same rationale, a click response procedure might perturb the coupling because cursor movements have no consequences in such a way that reaching a response option does not imply that cognitive processing has ended or even begun. In contrast, a hover response procedure might blur the coupling because participants avoid executing cursor movements early and freely due to fearing erroneous responses by accidently reaching the potentially wrong response option. Taking this angle, a hover response procedure might enhance the coupling, because cursor movements have consequences in such a way that reaching a response option demands that cognitive processing is ended. But also, here, again, the tighter constraints introduced by this procedure might in turn introduce additional processes in order to fulfill those which confounds the measurement.

Unfortunately, the dissociation between those intuitions cannot be resolved by the few recent studies that investigated the influence of the start and the response procedure on the quality of cursor movements. Therefore, we also cannot provide an ultimate answer to the question of which combination of design feature yields the most valid measurement of cognitive processing.

Instead, we can only conclude that design features are an important influence in mouse-tracking studies and that they can strongly affect the effects of theoretical interest.

### Is there heterogeneity in the field?

We conducted a systematic literature review including 114 full-text articles (consisting of 167 original experiments) on design features in mouse-tracking paradigms. By doing so, we obtained a comprehensive picture of the distribution of selected features (i.e., cursor speed, stimulus position, response box position, response procedure, response deadline, start procedure, sampling rate) applied in the last 15 years. Despite the identification of two prototypical mouse-tracking setup, we revealed substantial heterogeneity with respect to most examined design features.

### Lack of reporting?

While doing our systematic literature review, we identified a substantial lack of reporting: For only 1.81% of the reviewed experiments all eight design feature have been reported precisely, for 4.82% all eight design features were available at least indirectly (see Table 2). This leaves about 95% of studies which did not report all features that we had identified.

Specifically, we found that the cursor speed settings, the sampling rate, training, and time pressure were not fully reported in most of the reviewed articles. This finding probably reflects an unawareness in the community that those characteristics are crucial with respect to reproducibility (Goodman et al., 2016; Munafò et al., 2017), but also with respect to interpretation of the obtained results. Indeed, if we omit cursor speed settings from the set of desired features, the percentage of studies that report *all* seven other features goes up considerably, to 13.86%, and under the generous assumption that omitting response deadline and training details implies they were not employed, the percentage reporting all remaining five features rises to 43.37%. Unfortunately, we now know that these factors may matter considerably, and so fuller standards for reporting will help establish reproducible results. It makes a difference whether specific cursor trajectories occurred due to experimental manipulation or just a lack of training, or whether small movement of the computer mouse lead to a large cursor movement and was hence sufficient to reach the response boxes.

Even for the more striking characteristics of a mouse-tracking setup, we surprisingly observed several cases lacking proper reporting of, for instance, the response box position or the stimulus position (which is normally reported for experimental setups anyway; see Table 2). One possible explanation for this omission might simply be that many authors have something like a default setting in mind. Unfortunately, such a default has never been determined for mouse-tracking paradigms. Another possible explanation could be that the authors used ready-made software such as MouseTracker (Freeman & Ambady, 2010), and that they were thus not aware of the many different settings that are possible, and thought the ready-made software had been implemented the default settings which are unnecessary to report.^4^ It would be valuable to have these software packages provide guidelines about reporting their setup, especially under gold-standard reporting criteria (we return to this below).

### Can we trust the past?

So far, we identified relatively little consensus with respect to experimental design choices in combination with converging evidence that those design choices might influence the mapping between cognitive processing and cursor movements (see Fig. 1). Considering that we do not know yet which design features yield the best mapping, one might be tempted to mistrust the past mouse-tracking research due to a possibly flawed mapping between cursor movements and cognitive processing. However, such a conclusion would be premature, since past mouse-tracking research has reported several theoretically plausible and empirically robust effects. One traditional example from language is the typicality effect initially reported by R. Dale et al. (2007) and replicated several times (Kieslich & Henninger, 2017; Kieslich et al., 2020) by using different mouse-tracking setups. An example from action control would be the Simon effect, which proved to be very robust across studies, partially with varying mouse-tracking setups (Grage et al., 2019; Scherbaum & Dshemuchadse, 2020; Scherbaum et al., 2010). Another example from cognition is also the typicality effect in social perception (Freeman, Ambady, Rule, & Johnson, 2008). Therefore, any concern with reproducibility should focus on specific applications of the tracking technique, and distinguish between different aspects of the cognitive effects of interest, such as possible effect size and the mouse-tracking setup that has been used to measure it. In the analysis of cursor movements, it is commonly distinguished between discrete measures that summarize the trajectories in single values, and continuous measures that examine the temporal development of specific movement characteristics (Hehman et al., 2015; Kieslich et al., 2019; Scherbaum et al., 2010).

A large part of the past mouse-tracking literature focused on discrete measures (e.g., deflection; see Fig. 1), and it seems that for the occurrence of the larger effects the specific setup plays a minor role. However, recent studies suggest that the size of such effects might be inflated due to dissimilar distributed cursor movement trajectories incorporating considerably more extreme cursor trajectory shapes when a static start and a click response procedure was used in comparison to dynamic start and a hover response procedure (Grage et al., 2019; Kieslich et al., 2020).

For smaller effects on discrete measures, those findings could mean that the published results that had been obtained using one setup might not endure replications within another setup (Schoemann, Lüken, et al., 2019). It could also mean that choosing one procedure over the other increases the likelihood of producing publishable effects. Without proper reporting, one also runs into danger of inferring from different studies’ results on differences in the underlying processes, when instead, these differences might have been caused by different setups.

The same rationale but with the opposite direction holds for continuous measures (e.g., Scherbaum & Dshemuchadse, 2020; Sullivan, Hutcherson, Harris, & Rangel, 2015). Continuous trajectory measures assess the contribution of experimental variables on the cursor movement angle toward either response option over the time course. For example, Sullivan et al. (2015) estimated the effects of tastiness and healthfulness on time slices of trajectories during food choices, which indicated earlier engagement of the taste attribute in such decisions. However, recent results suggest that those effects decrease or even disappear in setups using a static rather than a dynamic start procedure (Scherbaum & Kieslich, 2018; Schoemann, Lüken, et al., 2019). Those findings agree with the intuition that for those measures it is critical that as much cognitive processing as possible is reflected in the cursor movements. Specifically, continuous regression approaches assume the same set of cognitive responses are occurring during the same portion of the trajectory across trials. To meet this assumption, the same portion of cognitive responding must be completed during the trajectory so that the cognition–trajectory alignment is consistent. Indeed, such alignment issues also arise in interpreting event-related neural potentials during cognitive processing (e.g., Jackson & Bolger, 2014).

In sum, our results and discussion do not recommend an easy comparison or synthesis of multiple mouse-tracking results across studies without taking the respective setups into account. As the cognitive effects of interest differ between various mouse-tracking setups, in meta-analyses, the specific procedures introduce additional heterogeneity which would impede a generalization beyond the studies included; instead, they would be limited to the range of mouse-tracking setups used in the included studies, which eventually impedes the accumulation of knowledge in mouse-tracking research. In this regard, it is important to note that we face not only a potential for Type I statistical error (false positives), but also Type II (false negatives): Choosing certain mouse-tracking procedures might also disrupt the cognition–movement mapping, and so *null effects* themselves may also emerge as a consequence.

Such a situation gives rise to the problems that are already well known in other areas of research—namely, publication bias (Francis, 2012; Renkewitz, Fuchs, & Fiedler, 2011; Schimmack, 2012; Simonsohn, Nelson, & Simmons, 2014) and questionable research practices (Banks, Rogelberg, Woznyj, Landis, & Rupp, 2016; John, Loewenstein, & Prelec, 2012). When effects can disappear due to choices of parameters that had not been reported in the original study, one might be tempted to attribute failure to myriad reasons and to question the results. The situation may also lead into methodological tweaking on a search for effects, opening up the problems inherent to p-hacking and the garden of forking paths (Simmons, Nelson, & Simonsohn, 2011; Wicherts et al., 2016). Agreed-upon standards for reporting design features would alleviate these issues and facilitate interpreting the outcome of statistical tests on discrete measures, like the deflection of a trajectory, in both rejecting or not rejecting the null hypothesis that some experimental factor modulates movement dynamics.

### How can we trust the future?

Turning from the past to the future, we identify two key challenges in the hope that mouse-tracking methods and findings can mature and evolve, especially in the service of achieving reproducibility and comparability.

First, in order to enhance reproducibility, the future challenge would be to develop reporting standards (Appelbaum et al., 2018) assuring that all relevant features of the mouse-tracking procedure (e.g., design features) are easily accessible, preferably in the form of text, but also in the form of figures or computer code. For eye tracking, another more traditional process-tracing method, this challenge has recently been taken by Fiedler, Schulte-Mecklenbeck, Renkewitz, and Orquin (2019), who uncovered a lack of reporting transparency and developed a minimal reporting standard “to promote the cultural shift towards openness and transparency in science to increased reproducibility, because precise, accurate and informative reporting is a prerequisite of reproducibility” (p. 74). As a matter of fact, mouse cursor tracking is very similar to eye tracking with respect to the required ingenuity: Researchers are faced with many decisions about the methods, materials, and procedures of which any decision may be more or less arbitrary and largely driven by the researcher’s idiosyncratic intuitions. Therefore, transparent reporting of these decisions is crucial to reproducibility and for others to judge the quality of the research (Goodman et al., 2016; Munafò et al., 2017; Nosek et al., 2015; Wicherts et al., 2016).

Here, we took a first step, and found that many details of experimental tasks are not available to the reader, sometimes even for some basic design features. The co6authors themselves have found this exercise to be quite useful, and have highlighted limitations in their own prior reporting. We suspect many readers may feel the same, and so the situation presents an exciting opportunity. Compiling an exhaustive list of all minimally required details of a mouse-tracking paradigm would be an exciting next step toward enhanced reproducibility. As a first step in this direction, based on our own hands-on experience from mouse-tracking research and from what we have learned from this review, we compiled a draft of minimal reporting standards, which we included in Appendix 2. It must be noted at this point, that open science practices, such as sharing of data and materials, are an important building block supporting reproducibility (Klein et al., 2018). Though the authors support and embrace these practices, they should be seen as a complement and cannot replace transparent reporting. The extraction of omitted parameters from published materials is a laborious procedure and practically not feasible—and in the case of closed-source software, even impossible. However, in light of the many options that mouse-tracking researchers face when designing their study, we see preregistration as an important practice (Nosek, Ebersole, DeHaven, & Mellor, 2018; Wagenmakers, Wetzels, Borsboom, van der Maas, & Kievit, 2012), which might profit from our second point: standards for mouse cursor tracking.

Second, in order to enhance comparability, the future challenge would be to develop a gold standard of mouse cursor tracking assuring that comparable methodologies are used in mouse-tracking experiments. In this regard, mouse cursor tracking is again similar to eye tracking, as methodological research suggests that the researchers’ many idiosyncratic design decisions may have unintended consequences for the data set and results (for eye tracking, see e.g., Orquin & Holmqvist, 2018). For mouse cursor tracking, we have reviewed recent methodological studies presenting such consequences (Grage et al., 2019; Kieslich et al., 2020; Scherbaum & Kieslich, 2018; Schoemann, Lüken, et al., 2019). In this regard, we see an urgent need to renew the already made calls toward the mouse-tracking community to agree on a standard mouse-tracking setup (Faulkenberry & Rey, 2014; Fischer & Hartmann, 2014; Hehman et al., 2015).

But could there be just one single gold standard mouse-tracking design over all domains and research questions? The diversity of questions mouse cursor tracking has been used to answer speaks against such an expectation. Certainly, researchers might always have good reasons for specific design decisions in mouse-tracking experiments. For instance, the complexity of the stimuli might be too high that a dynamic start procedure would ask too much of the participants with regard to processing while moving, such as in social dilemmas (e.g., Kieslich & Hilbig, 2014) or even in intertemporal choices (e.g., Calluso, Committeri, Pezzulo, Lepora, & Tosoni, 2015; O’Hora et al., 2016). However, for intertemporal choices, it has been demonstrated that the presentation of complex stimuli can be partitioned in such a way that even a dynamic start procedure can be feasible (e.g., Scherbaum, Frisch, & Dshemuchadse, 2018a, 2018b). In other instances, such as in the Simon task, where stimuli are easy to process, the usage of a dynamic start procedure might be required to capture relevant cognitive processing of competing response options rather than merely response selection behavior, which is crucial when applying mouse cursor tracking as a process-tracing method.

In any case, the development of one gold standard mouse-tracking setup presumes that we know how certain design choices affect the underlying cognitive processes as well as the validity of our measurement. Admittedly, we currently know too little in this respect and, hence, our call for standards in mouse-tracking research implies a call for more research to understand the relationship between cognitive processing and cursor movements. Explicit new reporting standards proposed here could help with this, too.

### The road ahead

In a way, our article mirrors the heterogeneity of the field: We aimed to summarize the current humble state of research on design features and summarized the current state in reporting practices on design features. Our results draw a picture in which the results and conclusions of mouse-tracking studies can be influenced by different design features. However, due to a lack of reporting of these features, the current picture is necessarily incomplete, and many questions must remain open. For instance, it remains unclear how the results from the three tasks generalize to other tasks, that is, how different processes and design features interact. To get the field on track into a future that offers replicability and reliable and interpretable results across many studies, we concluded that three things have to be done: First, the development of reporting standards that are embraced by the community (cf. Fiedler et al., 2019); second, systematic research on how design features affect mouse movements in different fields (cf. Baribault et al., 2018; Elson, 2019; Landy et al., 2020); and third, a gold standard—or improved standards—for how to implement the mouse-tracking procedure for different fields.

## Conclusion

A successful accumulation of knowledge in psychological science builds on valid inferences from observed measures onto cognitive processes. However, valid inferences do not come naturally; they crucially depend on methodological rigor as well as critical scrutinizing of the applied auxiliary assumptions. Sternberg (1969) scrutinized Donders’ (1868) *subtraction method* and developed his own *additive-factor method*, and hence substantially enhanced the measure of response time as a window into cognitive processing. Mouse cursor tracking is exquisitely sensitive to a range of design features and to enhance the potential of this method to explore cognition, we need to be clear about the implications of our design choices. Even though there may be preferred designs for specific phenomena, experimental designs should not only target the comparability between studies but also consider the validity of each single study. Hence, instead of one gold standard, different options should be available to researchers, though combined with the knowledge about the implications of each choice and with complete reporting to ensure that future research can reproduce and build on existing work.

### Supplementary Information

ESM 1 (DOCX 26.5 kb)

## Appendix 1

The synthesis of recent findings is based on reanalyzed original data from the four studies (Grage et al., 2019; Kieslich et al., 2020; Scherbaum & Kieslich, 2018; Schoemann, Lüken, et al., 2019). Initial data wrangling to standardize data structure and format (.csv) was conducted in MATLAB R2019b (The MathWorks, Natick, MA, USA). All analyses were conducted in R (R Core Team, 2018). The mouse cursor tracking related analyses were done using the *mousetrap* package (Kieslich & Henninger, 2017). Additionally, we used the *esc* package (Lüdecke, 2019), the *ordinal* package (Christensen, 2019), and the *metafor* package (Viechtbauer, 2010) to prepare and conduct the meta-analyses.

### Effect of the start procedure on the cognitive effect of interest

Before we turn to our main analysis with regard to the synthesis, we want to back up our reasoning from the main text, according which the start procedure introduced systematic variance to the respective cognitive effects in the included studies. In order to do so, we calculated the respective cognitive effect for each start condition separately (which unifies the analytical approach as the studies different with regard to how they statistically tested the influence of the start procedure). As the effect of the start procedure is not directly accessible in Grage et al. (2019), we followed the approach from Scherbaum and Kieslich (2018) and compared the data obtained with a static start procedure and a hover response procedure with the data from a formerly published experiment from our lab (viz. Scherbaum et al., 2010). The results show the coherent pattern that the cognitive effect of interest (e.g., Simon effect) is deemed to be smaller when a static start procedure is used as compared with a dynamic start procedure (see Table 3).

**Table 3:** Inferential results of the cognitive effect of interest for each study and start condition

Start procedure	Study	Effect	*N*	*t*	*p*	*d*_z_
Static	Grage2019	Simon effect	19	7.02	<.001	1.61
	Kieslich2019 Exp3	Typicality Effect	59	3.70	<.001	0.48
	Scherbaum2018	Simon effect	20	5.29	<.001	1.18
	Schoemann2019	SS vs. LL effect	35	1.05	.302	0.18
Dynamic	Grage2019	Simon effect	20	7.14	<.001	1.60
	Kieslich2019 Exp3	Typicality Effect	60	4.44	<.001	0.57
	Scherbaum2018	Simon effect	20	7.14	<.001	1.60
	Schoemann2019	SS vs. LL effect	36	1.83	.075	0.31

*Note.* The Simon effect is given by response-incongruent trials − response-congruent trials. The typicality effect is given by atypical trials − typical trials. The SS vs. LL effect is given by LL trials − SS trials. Please see our analysis script for the descriptive results for each within condition

### Effect of the start procedure on movement consistency within and across trials

We investigated the effect of the start procedure on movement consistency within and across trials based on two distinct indices: the continuous movement index (CMI) and the bimodality coefficient (BC). The CMI operates within trials and is given by the correlation of each empirical trajectory’s *y*-axis positions with those from a hypothetical constant and straight trajectory taken from the start to the end point of the respective empirical trajectory. We then averaged the CMI within participants for each start procedure (static vs. dynamic) separately. The BC operates across trials, is hence calculated on the participant level for each start procedure separately, and is based on the skewness and kurtosis of the distribution (Freeman & Dale, 2013; Pfister et al., 2013) of the trajectories’ *z*-scored (within participant), average deviations. Each trajectory’s average deviation, in turn, is defined as the average point distance (*x*-axis and y-axis position) of each empirical trajectory to a hypothetical constant and straight trajectory taken from the start to the end point of the respective empirical trajectory. After calculating those indices for each start procedure for each study separately, we statistically tested for differences between start conditions (static vs. dynamic) using two-tailed Student’s *t* tests for independent samples and calculated the effect sizes based on standardized mean differences (Siddaway, Wood, & Hedges, 2019). Table 4 gives an overview of our descriptive and inferential results; the corrected effect size index *g*_s_ was calculated following the formula given by Lakens (2013):

1 $$ {\mathrm{g}}_{\mathrm{s}}=\frac{{\overline{\mathrm{X}}}_1-{\overline{\mathrm{X}}}_2}{\sqrt{\frac{\left({n}_1-1\right){SD}_1^2+\left({n}_2-1\right){SD}_2^2}{n_1+{n}_2-2}}}\times \left(1-\frac{3}{4\left({n}_1+{n}_2\right)-9}\right). $$

**Table 4:** Descriptive and inferential results for each study and index

Index	Study	Static			Dynamic			Static vs. dynamic		
		*N*	*M*	*SD*	*N*	*M*	*SD*	*t*	*p*	*g*_s_
CMI	Grage2019	19	0.86	0.09	20	0.94	0.07	−3.14	<.01	−0.99
	Kieslich2019 Exp3	59	0.91	0.03	60	0.93	0.04	−2.54	<.05	−0.46
	Scherbaum2018	20	0.80	0.09	20	0.94	0.07	−5.24	<.01	−1.62
	Schoemann2019	35	0.86	0.10	36	0.95	0.05	−5.13	<.01	−1.22
BC	Grage2019	19	0.54	0.15	20	0.41	0.07	3.30	<.01	1.06
	Kieslich2019 Exp3	59	0.59	0.14	60	0.53	0.14	2.10	<.05	0.38
	Scherbaum2018	20	0.57	0.13	20	0.41	0.07	4.77	<.01	1.48
	Schoemann2019	35	0.65	0.13	36	0.40	0.14	7.71	<.01	1.81

In order to synthesize the four studies, we performed a fixed effects inverse variance meta-analysis on the obtained corrected effect sizes and estimated the average true effect in the four studies which is defined as follows:

2 $$ {\overline{\theta}}_{\mathrm{w}}=\sum \limits_{\mathrm{i}=1}^{\mathrm{k}}{w}_i{\theta}_i/\sum \limits_{\mathrm{i}=1}^{\mathrm{k}}{w}_i, $$

where ${\overline{\theta}}_w$ denotes the average true effect, and *θ*_*i*_ denotes the obtained corrected effect size *g*_s_, which is weighted by the inverse-variance, ${w}_i=1/{SD}_i^2$, for each study *i* in the set of *k* studies. The meta-analyses yielded significant average true effects of the start procedure on both the CMI (*b* = −0.87, *SE* = 0.13, *z* = −6.74, *p* < .001, 95% CI [−1.12, −0.62], BIC = 9.43) and the BC (*b* = 0.94, *SE* = 0.13, *z* = 7.13, *p* < .001, 95% CI [0.68, 1.19], BIC = 19.49). Heterogeneity was present in both models, *p*s < 0.02, but is neither estimated nor very important as “fixed-effects models provide perfectly valid inferences under heterogeneity, as long as one is restricting these inferences (i.e., the conclusions about the size of the average effect) to the set of studies included in the meta-analysis” (Viechtbauer, 2010, p. 4).

### Effect of the start procedure on the distribution of trajectory types

We investigated the effect of the start procedure on the distribution of trajectory types based on prototype mapping. Therefore, we followed the procedure described in Wulff et al. (2019) and mapped empirical trajectories to five trajectory prototypes—straight, curved, continuous change of mind (cCoM), discrete change of mind (dCoM), and double discrete change of mind (dCoM2)—as well as tested the equality of the obtained trajectory distributions across start procedures. Table 5 provides an overview of the descriptive and inferential results.

**Table 5:** Prototype mapping results. Proportions (in percentage) of empirical trajectories mapped to either of the five trajectory prototypes, as well as standardized (Pearson) residuals from tests of the equality of trajectory distributions across start procedures separately for each study

Start procedure	Study	Trajectory types (in %)				
		Straight	Curved	cCoM	dCoM	dCoM2
Proportions						
Static	Grage2019	56.85	32.27	9.31	1.38	0.19
	Kieslich2019 Exp3	58.01	19.24	13.74	6.73	2.27
	Scherbaum2018	65.96	25.32	7.21	1.38	0.13
	Schoemann2019	78.68	13.26	4.65	3.04	0.38
Dynamic	Grage2019	35.98	49.10	13.08	1.77	0.07
	Kieslich2019 Exp3	27.55	50.52	13.50	6.94	1.50
	Scherbaum2018	35.98	49.10	13.08	1.77	0.07
	Schoemann2019	36.35	53.06	9.62	0.86	0.10
Std. residuals						
Static	Grage2019	18.16	−16.08	−6.85	−1.87	1.97
	Kieslich2019 Exp3	7.61	−8.64	0.11	−0.13	0.92
	Scherbaum2018	25.43	−23.66	−11.19	−1.87	1.15
	Schoemann2019	34.54	−42.65	−11.51	9.68	3.44
Dynamic	Grage2019	−23.66	18.30	7.80	2.13	−2.24
	Kieslich2019 Exp3	−7.57	8.59	−0.11	0.13	−0.92
	Scherbaum2018	−25.61	23.83	11.26	1.88	−1.16
	Schoemann2019	−34.34	42.41	11.44	−9.62	−3.42

*Note.* Proportions are based on the minimal Euclidian distance for each space-normalized empirical trajectory to either of the five trajectory prototypes (Wulff et al., 2019). Standardized (Pearson) residuals ($stdres=\frac{obs-\mathit{\exp}}{\sqrt{V}}$, with *V* being the residual cell variance) are based on χ^2^ tests of stochastic independence between the frequency distributions of the static and dynamic start procedure, separately for each study, 265.37 ≤ χ^2^(4)s ≤ 6462.40, *p*s < .001; the residuals denote how strongly the cells of the contingency table deviate from the expected frequency, and hence, which cells drive the result of the χ^2^ test

We then ordered the trajectory types with respect to the degree of conflict/response competition (i.e., competing activation of the options; see Fig. 1) they should map (Wulff et al., 2019) and determined the effect of the start procedure via an ordinal mixed regression, for each study separately (see Table 6). The results of the ordinal mixed regressions all point to the same conclusion: The start conditions produced trajectories consistent with significantly different degrees of response competition—namely, more competition in the dynamic start condition.

**Table 6:** Ordinal mixed regression results

Study	*b*	*SE*	*z*	*p*
Grage2019	1.03	0.39	2.66	<.01
Kieslich2019 Exp3	0.92	0.18	5.07	<.01
Scherbaum2018	1.31	0.30	4.41	<.01
Schoemann2019	1.92	0.27	7.08	<.01

*Note.* Parameter are extracted from an ordinal mixed-regression model on the degree of competition between the response options (straight < curved < cCoM < dCoM < dCoM2). The start procedure was dummy coded (−0.5 = static; 0.5 = dynamic) and included as a fixed effect with random intercept and fixed slope for participants. The model was fitted using a logit link function and the Laplace approximation

In order to synthesize the four studies with regard to the results of the ordinal mixed regression, we again performed a fixed effects inverse variance meta-analysis on the obtained unstandardized regression coefficients and estimated the average true effect. Estimating the average true effect based on regression coefficients seems to be valid approach since the coefficients resulted from the same statistical models (Becker & Wu, 2007; Peterson & Brown, 2005). The meta-analyses yielded significant average true effects of the start procedure (*b* = 1.22, *SE* = 0.13, *z* = 9.62, *p* < .001, 95% CI [0.97, 1.47]).

## Appendix 2

### Minimal reporting standards

Here, we present a first draft of minimal reporting standards for mouse cursor tracking (inspired by Fiedler et al., 2019).

**Table:**

**Method**	
***Description of the mouse-tracking device***	
Model (e.g., M110 Silent) [Computer mouse model]	
Producer/brand (e.g., Logitech) [Computer mouse producer]	
Type (e.g., laser or optical with 1000 dpi and 125 Hz, wireless-2.4GHz-connection) [Computer mouse sensor technology including resolution and sampling rate, Computer mouse connection technology]	
Surface (e.g., mousepad) [Computer mouse surface]	
***Description of the monitor***	
Model (e.g., BenQ Senseye 3) [Monitor model]	
Producer (e.g., BenQ) [Monitor producer]	
Resolution [Screen resolution]	
Size [Screen size]	
***Description of the Software***	
Software settings for the computer mouse and the resulting hand/cursor movement ratio (e.g., cursor speed, acceleration) [Cursor settings]	
Software used to record the mouse-tracking data [Software record]	
Stimulus presentation software [Software present]	
**Material**	
Absolute size of start box and its content [Start box size]	
Absolute size of response boxes and its contents [Response boxes size]	
Absolute distance between response boxes [Response box distance]	
Absolute distance between response boxes and start box [Start box distance]	
Absolute size of stimulus [Stimulus size]	
**Procedure**	
Hand used (and handedness of participants) [Handedness]	
Practicing trials [Training]	
Procedure-related feedback [Feedback]	
Awareness of participants [Awareness]	
Procedure of trial start [Start procedure incl. Movement initiation deadline]	
Procedure of response indication [Response procedure incl. Response deadline]	
Duration of stimulus presentation [Stimulus duration]	
Counter balancing of response boxes [Response box mapping]	
Location of response boxes [Response box position]	
Location of start box [Start box position]	
Location of stimulus [Stimulus position]	
Number of trials [Trials]	
Settings and locations where data was collected [Location]	
**Results**	
***Data quality***	
Proportion of trials excluded for the analysis [Exclusion trial]	
Reasons for exclusion [Exclusion reason]	
Number of participants excluded from the analysis [Exclusion participants]	
Quality threshold for data exclusion [Exclusion quality]	
Sampling rate of the data [Sampling rate]	
***Dependent measures***	
Normalization method for data [Normalization]	
Indexation method for discrete measures [Indexation]	
Additional transformation of the data [Transformation]	

## Footnotes

1 Cursor speed is best defined as the mapping of movement in real space to the movement on the screen. Importantly, assessing cursor speed requires switching off any nonlinear factors on the driver settings of the computer mouse. Then, cursor speed can be assessed by moving the mouse in real space (e.g., for 10 cm) and measuring the moved distance in pixels on screen (e.g., with the Pixelruler software; www.pixelruler.de; M. Rosenbaum, Ratzeburg, Germany). This can then be converted, for example, to pixels per centimeter of movement.

2 Stimuli have (contextual) meanings in, for example, economic studies using Mouselab (Bettman, Johnson, & Payne, 1990) or when studying cursor movements on websites, where cursor movements serve as a proxy for attentional/perceptual processes.

3 This was the study by Scherbaum and Kieslich (2018) described above. It was excluded since it intentionally varied the start procedure.

4 In the case of MouseTracker (Freeman & Ambady, 2010), the software does not work with specific default values with respect to the investigated design features. Instead, the user needs to begin with the source file of a sample experiment being the only starting point when designing mouse-tracking experiments in MouseTracker. Hence, the parameter in this source file might work like default values. In the sample experiment of a task with two response option—to which it is also referred in the software documentary—the authors of MouseTracker implemented a static start procedure with a movement initiation deadline of 1,000 ms and a click response procedure; they response boxes were located in the upper corners of the screen, and the stimuli were presented in the center of the screen; the cursor speed is not mentioned.
