# Supplementary material for: Using mouse cursor tracking to investigate online cognition: Preserving methodological ingenuity while moving toward reproducible science
Source: Psychon Bull Rev. 2020 Dec 14;28(3):766–87. doi: 10.3758/s13423-020-01851-3 (PMC8219569; doi:10.3758/s13423-020-01851-3)
Supplement: Supplementary file 1 — (DOCX 26.5 kb) [file 13423_2020_1851_MOESM1_ESM.docx]

Using mouse cursor tracking to investigate online cognition: Preserving methodological ingenuity while moving toward reproducible science

— Supplementary material —

Martin Schoemann

Technische Universität Dresden, Germany & Aarhus University, Denmark

Denis O’Hora

National University of Ireland, Galway, Ireland

Rick Dale

University of California, Los Angeles, USA

Stefan Scherbaum

Technische Universität Dresden, Germany

List of references yielding data for the systematic review

Abney, D. H., McBride, D. M., Conte, A. M., & Vinson, D. W. (2015). Response dynamics in prospective memory. *Psychonomic Bulletin and Review*, *22*(4), 1020–1028. https://doi.org/10.3758/s13423-014-0771-6

Aczel, B., Szaszi, B., & Kieslich, P. J. (2018). Supplemental Material for Is Action Execution Part of the Decision-Making Process? An Investigation of the Embodied Choice Hypothesis. *Journal of Experimental Psychology: Learning, Memory, and Cognition*, *44*(6), 918–926. https://doi.org/10.1037/xlm0000484.supp

Barca, L., Benedetti, F., & Pezzulo, G. (2016). The effects of phonological similarity on the semantic categorisation of pictorial and lexical stimuli: evidence from continuous behavioural measures. *Journal of Cognitive Psychology*, *28*(2), 159–170. https://doi.org/10.1080/20445911.2015.1101117

Barca, L., & Pezzulo, G. (2015). Tracking second thoughts: Continuous and discrete revision processes during visual lexical decision. *PLoS ONE*, *10*(2), 1–14. https://doi.org/10.1371/journal.pone.0116193

Barca, L., Pezzulo, G., Ouellet, M., & Ferrand, L. (2017). Dynamic lexical decisions in French: Evidence for a feedback inconsistency effect. *Acta Psychologica*, *180*(April 2016), 23–32. https://doi.org/10.1016/j.actpsy.2017.08.005

Bartolotti, J., & Marian, V. (2012). Language Learning and Control in Monolinguals and Bilinguals. *Cognitive Science*, *36*(6), 1129–1147. https://doi.org/10.1111/j.1551-6709.2012.01243.x

Blazej, L. J., & Cohen-Goldberg, A. M. (2015). Can we hear morphological complexity before words are complex? *Journal of Experimental Psychology: Human Perception and Performance*, *41*(1), 50–68. https://doi.org/10.1037/a0038509

Brambilla, M., Biella, M., & Freeman, J. B. (2018). The influence of visual context on the evaluation of facial trustworthiness. *Journal of Experimental Social Psychology*, *78*(September 2017), 34–42. https://doi.org/10.1016/j.jesp.2018.04.011

Bruhn, P. (2013). Emergence of spontaneous anticipatory hand movements in a probabilistic environment. *Advances in Cognitive Psychology*, *9*(2), 62–73. https://doi.org/10.2478/vl0053-008-0132-y

Bundt, C., Ruitenberg, M. F. L., Abrahamse, E. L., & Notebaert, W. (2018). Early and late indications of item-specific control in a Stroop mouse tracking study. *PLOS ONE*, *13*(5), e0197278. https://doi.org/10.1371/journal.pone.0197278

Buttlar, B., & Walther, E. (2018). Measuring the meat paradox: How ambivalence towards meat influences moral disengagement. *Appetite*, *128*(December 2017), 152–158. https://doi.org/10.1016/j.appet.2018.06.011

Calcagnì, A., & Lombardi, L. (2014). Dynamic Fuzzy Rating Tracker (DYFRAT): a novel methodology for modeling real-time dynamic cognitive processes in rating scales. *Applied Soft Computing*, *24*, 948–961. https://doi.org/10.1016/j.asoc.2014.08.049

Calluso, C., Committeri, G., Pezzulo, G., Lepora, N., & Tosoni, A. (2015). Analysis of hand kinematics reveals inter-individual differences in intertemporal decision dynamics. *Experimental Brain Research*, *233*(12), 3597–3611. https://doi.org/10.1007/s00221-015-4427-1

Calluso, C., Saulin, A., Baumgartner, T., & Knoch, D. (2018). Distinct Patterns of Cognitive Conflict Dynamics in Promise Keepers and Promise Breakers. *Frontiers in Psychology*, *9*(JUN), 1–12. https://doi.org/10.3389/fpsyg.2018.00939

Capellini, R., Sacchi, S., Ricciardelli, P., & Actis-Grosso, R. (2016). Social Threat and Motor Resonance: When a Menacing Outgroup Delays Motor Response. *Frontiers in Psychology*, *7*(November), 1–11. https://doi.org/10.3389/fpsyg.2016.01697

Carpenter, S. K., Lohse, K. R., Healy, A. F., Bourne, L. E., & Clegg, B. A. (2013). External focus of attention improves performance in a speeded aiming task. *Journal of Applied Research in Memory and Cognition*, *2*(1), 14–19. https://doi.org/10.1016/j.jarmac.2012.11.002

Carraro, L., Castelli, L., & Negri, P. (2016). The hand in motion of liberals and conservatives reveals the differential processing of positive and negative information. *Acta Psychologica*, *168*, 78–84. https://doi.org/10.1016/j.actpsy.2016.04.006

Cloutier, J., Freeman, J. B., & Ambady, N. (2014). Investigating the Early Stages of Person Perception: The Asymmetry of Social Categorization by Sex vs. Age. *PLoS ONE*, *9*(1), e84677. https://doi.org/10.1371/journal.pone.0084677

Coco, M. I., & Duran, N. D. (2016). When expectancies collide: Action dynamics reveal the interaction between stimulus plausibility and congruency. *Psychonomic Bulletin & Review*, *23*(6), 1920–1931. https://doi.org/10.3758/s13423-016-1033-6

Cranford, E. A., & Moss, J. (2018). Mouse-tracking evidence for parallel anticipatory option evaluation. *Cognitive Processing*, *19*(3), 327–350. https://doi.org/10.1007/s10339-017-0851-4

Dale, R., & Duran, N. D. (2011). The Cognitive Dynamics of Negated Sentence Verification. *Cognitive Science*, *35*(5), 983–996. https://doi.org/10.1111/j.1551-6709.2010.01164.x

Dale, R., Kehoe, C., & Spivey, M. J. (2007). Graded motor responses in the time course of categorizing atypical exemplars. *Memory & Cognition*, *35*(1), 15–28. https://doi.org/10.3758/BF03195938

Damiam, M. F., Ye, W., Oh, M., & Yang, S. (2019). Bilinguals as “experts”? Comparing performance of mono- to bilingual individuals via a mousetracking paradigm. *Bilingualism: Language and Cognition*, *22*(5), 1176–1193. https://doi.org/10.1017/S1366728918000901

Dignath, D., Pfister, R., Eder, A. B., Kiesel, A., & Kunde, W. (2014). Something in the way she moves—movement trajectories reveal dynamics of self-control. *Psychonomic Bulletin & Review*, *21*(3), 809–816. https://doi.org/10.3758/s13423-013-0517-x

Dshemuchadse, M., Grage, T., & Scherbaum, S. (2015). Action dynamics reveal two types of cognitive flexibility in a homonym relatedness judgment task. *Frontiers in Psychology*, *6*, 1244. https://doi.org/10.3389/fpsyg.2015.01244

Dshemuchadse, M., Scherbaum, S., & Goschke, T. (2013). How decisions emerge: Action dynamics in intertemporal decision making. *Journal of Experimental Psychology: General*, *142*(1), 93–100. https://doi.org/10.1037/a0028499

Duran, N. D., Nicholson, S. P., & Dale, R. (2017). The hidden appeal and aversion to political conspiracies as revealed in the response dynamics of partisans. *Journal of Experimental Social Psychology*, *73*(June 2016), 268–278. https://doi.org/10.1016/j.jesp.2017.07.008

Easey, K. E., Catling, J. C., Kent, C., Crouch, C., Jackson, S., Munafò, M. R., & Attwood, A. S. (2018). State anxiety and information processing: A 7.5% carbon dioxide challenge study. *Psychonomic Bulletin and Review*, *25*(2), 732–738. https://doi.org/10.3758/s13423-017-1413-6

Faulkenberry, T. J. (2016). Testing a direct mapping versus competition account of response dynamics in number comparison †. *Journal of Cognitive Psychology*, *28*(7), 825–842. https://doi.org/10.1080/20445911.2016.1191504

Faulkenberry, T. J., Cruise, A., Lavro, D., & Shaki, S. (2016). Response trajectories capture the continuous dynamics of the size congruity effect. *Acta Psychologica*, *163*, 114–123. https://doi.org/10.1016/j.actpsy.2015.11.010

Faulkenberry, T. J., Cruise, A., & Shaki, S. (2017). Reversing the Manual Digit Bias in Two-Digit Number Comparison. *Experimental Psychology*, *64*(3), 191–204. https://doi.org/10.1027/1618-3169/a000365

Faulkenberry, T. J., Cruise, A., & Shaki, S. (2018). Task instructions modulate unit–decade binding in two-digit number representation. *Psychological Research*, *0*(0), 1–16. https://doi.org/10.1007/s00426-018-1057-9

Faulkenberry, T. J., Montgomery, S. A., & Tennes, S.-A. N. (2015). Response trajectories reveal the temporal dynamics of fraction representations. *Acta Psychologica*, *159*, 100–107. https://doi.org/10.1016/j.actpsy.2015.05.013

Flumini, A., Barca, L., Borghi, A. M., & Pezzulo, G. (2015). How do you hold your mouse? Tracking the compatibility effect between hand posture and stimulus size. *Psychological Research*, *79*(6), 928–938. https://doi.org/10.1007/s00426-014-0622-0

Freeman, J. B. (2014). Abrupt category shifts during real-time person perception. *Psychonomic Bulletin and Review*, *21*(1), 85–92. https://doi.org/10.3758/s13423-013-0470-8

Freeman, J. B., & Ambady, N. (2010). MouseTracker: Software for studying real-time mental processing using a computer mouse-tracking method. *Behavior Research Methods*, *42*(1), 226–241. https://doi.org/10.3758/BRM.42.1.226

Freeman, J. B., Ma, Y., Han, S., & Ambady, N. (2013). Influences of culture and visual context on real-time social categorization. *Journal of Experimental Social Psychology*, *49*(2), 206–210. https://doi.org/10.1016/j.jesp.2012.10.015

Freeman, J. B., Pauker, K., Apfelbaum, E. P., & Ambady, N. (2010). Continuous dynamics in the real-time perception of race. *Journal of Experimental Social Psychology*, *46*(1), 179–185. https://doi.org/10.1016/j.jesp.2009.10.002

Freeman, J. B., Pauker, K., & Sanchez, D. T. (2016). A Perceptual Pathway to Bias. *Psychological Science*, *27*(4), 502–517. https://doi.org/10.1177/0956797615627418

Frisch, S., Dshemuchadse, M., Görner, M., Goschke, T., & Scherbaum, S. (2015). Unraveling the sub-processes of selective attention: insights from dynamic modeling and continuous behavior. *Cognitive Processing*, *16*(4), 377–388. https://doi.org/10.1007/s10339-015-0666-0

Galati, A., Dale, R., & Duran, N. D. (2019). Social and configural effects on the cognitive dynamics of perspective-taking. *Journal of Memory and Language*, *104*(August 2018), 1–24. https://doi.org/10.1016/j.jml.2018.08.007

Georgii, C., Goldhofer, P., Meule, A., Richard, A., & Blechert, J. (2017). Food craving, food choice and consumption: The role of impulsivity and sham-controlled tDCS stimulation of the right dlPFC. *Physiology and Behavior*, *177*(November 2016), 20–26. https://doi.org/10.1016/j.physbeh.2017.04.004

Gill, M. J., & Ungson, N. D. (2018). How much blame does he truly deserve? Historicist narratives engender uncertainty about blameworthiness, facilitating motivated cognition in moral judgment. *Journal of Experimental Social Psychology*, *77*(March), 11–23. https://doi.org/10.1016/j.jesp.2018.03.008

Gürçay, B., & Baron, J. (2017). Challenges for the sequential two-system model of moral judgement. *Thinking and Reasoning*, *23*(1), 49–80. https://doi.org/10.1080/13546783.2016.1216011

Ha, O.-R., Bruce, A. S., Pruitt, S. W., Cherry, J. B. C., Smith, T. R., Burkart, D., … Lim, S.-L. (2016). Healthy eating decisions require efficient dietary self-control in children: A mouse-tracking food decision study. *Appetite*, *105*, 575–581. https://doi.org/10.1016/j.appet.2016.06.027

Hartmann, M. (2017). Non-musicians also have a piano in the head: evidence for spatial–musical associations from line bisection tracking. *Cognitive Processing*, *18*(1), 75–80. https://doi.org/10.1007/s10339-016-0779-0

Haslbeck, J. M. B., Wood, G., & Witte, M. (2016). Article Commentary: Temporal dynamics of number-space interaction in line bisection: Comment on Cleland and Bull (2015). *Quarterly Journal of Experimental Psychology*, *69*(6), 1239–1242. https://doi.org/10.1080/17470218.2015.1095773

Hehman, E., Ingbretsen, Z. A., & Freeman, J. B. (2014). The neural basis of stereotypic impact on multiple social categorization. *NeuroImage*, *101*, 704–711. https://doi.org/10.1016/j.neuroimage.2014.07.056

Hermens, F. (2018). When do arrows start to compete? A developmental mouse-tracking study. *Acta Psychologica*, *182*(November 2017), 177–188. https://doi.org/10.1016/j.actpsy.2017.11.015

Hermens, F., Bindemann, M., & Mike Burton, A. (2017). Responding to social and symbolic extrafoveal cues: cue shape trumps biological relevance. *Psychological Research*, *81*(1), 24–42. https://doi.org/10.1007/s00426-015-0733-2

Hindy, N. C., Hamilton, R., Houghtling, A. S., Coslett, H. B., & Thompson-Schill, S. L. (2009). Computer-Mouse Tracking Reveals TMS Disruptions of Prefrontal Function During Semantic Retrieval. *Journal of Neurophysiology*, *102*(6), 3405–3413. https://doi.org/10.1152/jn.00516.2009

Huette, S. (2016). Putting context into context: sources of context and a proposed mechanism for linguistic negation. *Language, Cognition and Neuroscience*, *31*(8), 1000–1014. https://doi.org/10.1080/23273798.2016.1161807

Huette, S., & McMurray, B. (2010). Continuous dynamics of color categorization. *Psychonomic Bulletin & Review*, *17*(3), 348–354. https://doi.org/10.3758/PBR.17.3.348

Incera, S., & McLennan, C. T. (2016). Mouse tracking reveals that bilinguals behave like experts. *Bilingualism*, *19*(3), 610–620. https://doi.org/10.1017/S1366728915000218

Incera, S., & McLennan, C. T. (2018). The time course of within and between-language interference in bilinguals. *International Journal of Bilingualism*, *22*(1), 88–99. https://doi.org/10.1177/1367006916644688

Incera, S., & McLennan, C. T. (2018). Bilingualism and age are continuous variables that influence executive function. *Aging, Neuropsychology, and Cognition*, *25*(3), 443–463. https://doi.org/10.1080/13825585.2017.1319902

Incera, S., McLennan, C. T., Stronsick, L. M., & Zetzer, E. E. (2019). Is tuba masculine or feminine? The timing of grammatical gender. *Mind and Language*, *34*(5), 667–680. https://doi.org/10.1111/mila.12223

Iodice, P., Calluso, C., Barca, L., Bertollo, M., Ripari, P., & Pezzulo, G. (2017). Fatigue increases the perception of future effort during decision making. *Psychology of Sport and Exercise*, *33*, 150–160. https://doi.org/10.1016/j.psychsport.2017.08.013

Janczyk, M., Pfister, R., & Kunde, W. (2013). Mice move smoothly: irrelevant object variation affects perception, but not computer mouse actions. *Experimental Brain Research*, *231*(1), 97–106. https://doi.org/10.1007/s00221-013-3671-5

Kang, S. H. K., Eglington, L. G., & Yap, M. J. (2018). Forward versus backward semantic priming: What movement dynamics during lexical decision reveal. *Quarterly Journal of Experimental Psychology*, *71*(7), 1506–1511. https://doi.org/10.1177/1747021818775051

Kawakami, N., & Miura, E. (2019). Tracking hand movements captures the response dynamics of the evaluative priming effect. *Cognition and Emotion*, *33*(3), 452–465. https://doi.org/10.1080/02699931.2018.1483897

Kieslich, P. J., & Henninger, F. (2017). Mousetrap: An integrated, open-source mouse-tracking package. *Behavior Research Methods*, *49*(5), 1652–1667. https://doi.org/10.3758/s13428-017-0900-z

Kieslich, P. J., & Hilbig, B. E. (2014). Cognitive conflict in social dilemmas: An analysis of response dynamics. *Judgment and Decision Making*, *9*(6), 510–522.

Koop, G. J. (2013). An assessment of the temporal dynamics of moral decisions. *Judgment and Decision Making*, *8*(5), 527–539.

Koop, G. J., & Criss, A. H. (2016). The response dynamics of recognition memory: Sensitivity and bias. *Journal of Experimental Psychology: Learning, Memory, and Cognition*, *42*(5), 671–685. https://doi.org/10.1037/xlm0000202

Lazerus, T., Ingbretsen, Z. A., Stolier, R. M., Freeman, J. B., & Cikara, M. (2016). Positivity bias in judging ingroup members’ emotional expressions. *Emotion*, *16*(8), 1117–1125. https://doi.org/10.1037/emo0000227

Leontyev, A., Sun, S., Wolfe, M., & Yamauchi, T. (2018). Augmented Go/No-Go task: Mouse cursor motion measures improve ADHD symptom assessment in healthy college students. *Frontiers in Psychology*, *9*(APR), 1–14. https://doi.org/10.3389/fpsyg.2018.00496

Li, H., & Wang, F. (2019). Real-time measurement of wise personality cognition: Evidence from mouse tracking. *Current Psychology*, *38*(6), 1748–1762. https://doi.org/10.1007/s12144-017-9732-3

Liao, M.-J., & Wang, S.-H. (2015). Exploring the role of stimulus code–response modality compatibility on the spatial Stroop effect. *Ergonomics*, *58*(8), 1372–1387. https://doi.org/10.1080/00140139.2015.1005169

Lim, S. L., Penrod, M. T., Ha, O. R., Bruce, J. M., & Bruce, A. S. (2018). Calorie Labeling Promotes Dietary Self-Control by Shifting the Temporal Dynamics of Health- and Taste-Attribute Integration in Overweight Individuals. *Psychological Science*, *29*(3), 447–462. https://doi.org/10.1177/0956797617737871

Lin, Y. C., & Lin, P. Y. (2016). Mouse tracking traces the “Camrbidge Unievrsity” effects in monolingual and bilingual minds. *Acta Psychologica*, *167*, 52–62. https://doi.org/10.1016/j.actpsy.2016.04.001

Lin, Y.-C., Bangert, A. S., & Schwartz, A. I. (2015). The devil is in the details of hand movement: Visualizing transposed-letter effects in bilingual minds. *The Mental Lexicon*, *10*(3), 364–389. https://doi.org/10.1075/ml.10.3.03lin

Loy, J. E., Rohde, H., & Corley, M. (2017). Effects of Disfluency in Online Interpretation of Deception. *Cognitive Science*, *41*, 1434–1456. https://doi.org/10.1111/cogs.12378

Martens, M. A., Hasinski, A. E., Andridge, R. R., & Cunningham, W. A. (2012). Continuous cognitive dynamics of the evaluation of trustworthiness in Williams syndrome. *Frontiers in Psychology*, *3*(JUN), 1–9. https://doi.org/10.3389/fpsyg.2012.00160

Mattek, A. M., Whalen, P. J., Berkowitz, J. L., & Freeman, J. B. (2016). Differential effects of cognitive load on subjective versus motor responses to ambiguously valenced facial expressions. *Emotion*, *16*(6), 929–936. https://doi.org/10.1037/emo0000148

Monaro, M., Gamberini, L., & Sartori, G. (2017). The detection of faked identity using unexpected questions and mouse dynamics. *PLOS ONE*, *12*(5), e0177851. https://doi.org/10.1371/journal.pone.0177851

Monaro, M., Toncini, A., Ferracuti, S., Tessari, G., Vaccaro, M. G., De Fazio, P., … Sartori, G. (2018). The detection of malingering: A new tool to identify made-up depression. *Frontiers in Psychiatry*, *9*(JUN), 1–12. https://doi.org/10.3389/fpsyt.2018.00249

Morett, L. M., & MacWhinney, B. (2013). Syntactic transfer in English-speaking Spanish learners. *Bilingualism*, *16*(1), 132–151. https://doi.org/10.1017/S1366728912000107

Papesh, M. H. (2015). Just out of reach: On the reliability of the action-sentence compatibility effect. *Journal of Experimental Psychology: General*, *144*(6), e116–e141. https://doi.org/10.1037/xge0000125

Papesh, M. H., & Goldinger, S. D. (2012). Memory in motion: Movement dynamics reveal memory strength. *Psychonomic Bulletin & Review*, *19*, 906–913. https://doi.org/10.3758/s13423-012-0281-3

Quétard, B., Quinton, J. C., Colomb, M., Pezzulo, G., Barca, L., Izaute, M., … Mermillod, M. (2015). Combined effects of expectations and visual uncertainty upon detection and identification of a target in the fog. *Cognitive Processing*, *16*, 343–348. https://doi.org/10.1007/s10339-015-0673-1

Quétard, B., Quinton, J. C., Mermillod, M., Barca, L., Pezzulo, G., Colomb, M., & Izaute, M. (2016). Differential effects of visual uncertainty and contextual guidance on perceptual decisions: Evidence from eye and mouse tracking in visual search. *Journal of Vision*, *16*(11), 28. https://doi.org/10.1167/16.11.28

Quinton, J.-C., & Smeding, A. (2015). Dynamic competition and binding of concepts through time and space. *Cognitive Processing*, *16*(S1), 349–353. https://doi.org/10.1007/s10339-015-0674-0

Ratcliff, R. (2018). Decision making on spatially continuous scales. *Psychological Review*, *125*(6), 888–935. https://doi.org/10.1037/rev0000117

Ruitenberg, M. F. L., Abrahamse, E. L., Santens, P., & Notebaert, W. (2019). The effect of dopaminergic medication on conflict adaptation in Parkinson’s disease. *Journal of Neuropsychology*, *13*(1), 121–135. https://doi.org/10.1111/jnp.12131

Ruitenberg, M. F. L., Duthoo, W., Santens, P., Seidler, R. D., Notebaert, W., & Abrahamse, E. L. (2016). Sequence learning in Parkinson’s disease: Focusing on action dynamics and the role of dopaminergic medication. *Neuropsychologia*, *93*(September), 30–39. https://doi.org/10.1016/j.neuropsychologia.2016.09.027

Scherbaum, S., Dshemuchadse, M., Fischer, R., & Goschke, T. (2010). How decisions evolve: The temporal dynamics of action selection. *Cognition*, *115*(3), 407–416. https://doi.org/10.1016/j.cognition.2010.02.004

Scherbaum, S., Dshemuchadse, M., Leiberg, S., & Goschke, T. (2013). Harder than expected: Increased conflict in clearly disadvantageous delayed choices in a computer game. *PLoS ONE*, *8*(11). https://doi.org/10.1371/journal.pone.0079310

Scherbaum, S., Frisch, S., & Dshemuchadse, M. (2018). A bird in the hand isn’t good for long: Action dynamics reveal short-term choice impulses in intertemporal choices. *Experimental Psychology*, *65*(1), 23–31. https://doi.org/10.1027/1618-3169/a000385

Scherbaum, S., Frisch, S., & Dshemuchadse, M. (2018). Step by step: Harvesting the dynamics of delay discounting decisions. *Quarterly Journal of Experimental Psychology (2006)*, *71*(4), 949–964. https://doi.org/10.1080/17470218.2017.1307863

Scherbaum, S., Frisch, S., Dshemuchadse, M., Rudolf, M., & Fischer, R. (2018). The test of both worlds: identifying feature binding and control processes in congruency sequence tasks by means of action dynamics. *Psychological Research*, *82*(2), 337–352. https://doi.org/10.1007/s00426-016-0823-9

Scherbaum, S., Gottschalk, C., Dshemuchadse, M., & Fischer, R. (2015). Action dynamics in multitasking: the impact of additional task factors on the execution of the prioritized motor movement. *Frontiers in Psychology*, *6*(July), 1–8. https://doi.org/10.3389/fpsyg.2015.00934

Scherbaum, S., & Kieslich, P. J. (2018). Stuck at the starting line: How the starting procedure influences mouse-tracking data. *Behavior Research Methods*, *50*(5), 2097–2110. https://doi.org/10.3758/s13428-017-0977-4

Schneider, I. K., van Harreveld, F., Rotteveel, M., Topolinski, S., van der Pligt, J., Schwarz, N., & Koole, S. L. (2015). The path of ambivalence: tracing the pull of opposing evaluations using mouse trajectories. *Frontiers in Psychology*, *6*(July), 1–12. https://doi.org/10.3389/fpsyg.2015.00996

Smeding, A., Quinton, J., Lauer, K., Barca, L., & Pezzulo, G. (2016). Supplemental Material for Tracking and Simulating Dynamics of Implicit Stereotypes: A Situated Social Cognition Perspective. *Journal of Personality and Social Psychology*, *111*(6), 817–834. https://doi.org/10.1037/pspa0000063.supp

Smith, J. R., Treat, T. A., Farmer, T. A., & McMurray, B. (2018). Dynamic competition account of men’s perceptions of women’s sexual interest. *Cognition*, *174*(December 2017), 43–54. https://doi.org/10.1016/j.cognition.2017.12.016

Squires, L. (2014). Processing, Evaluation, Knowledge. *Journal of English Linguistics*, *42*(2), 144–172. https://doi.org/10.1177/0075424214526057

Stillman, P. E., Medvedev, D., & Ferguson, M. J. (2017). Resisting Temptation: Tracking How Self-Control Conflicts Are Successfully Resolved in Real Time. *Psychological Science*, *28*(9), 1240–1258. https://doi.org/10.1177/0956797617705386

Sullivan, N., Hutcherson, C., Harris, A., & Rangel, A. (2015). Dietary Self-Control Is Related to the Speed With Which Attributes of Healthfulness and Tastiness Are Processed. *Psychological Science*, *26*(2), 122–134. https://doi.org/10.1177/0956797614559543

Szaszi, B., Palfi, B., Szollosi, A., Kieslich, P. J., & Aczel, B. (2018). Thinking dynamics and individual differences: Mouse-tracking analysis of the denominator neglect task. *Judgment and Decision Making*, *13*(1), 23–32. Retrieved from http://journal.sjdm.org/17/17818/jdm17818.pdf

Tomlinson, J. M., Bailey, T. M., & Bott, L. (2013). Possibly all of that and then some: Scalar implicatures are understood in two steps. *Journal of Memory and Language*, *69*(1), 18–35. https://doi.org/10.1016/j.jml.2013.02.003

Tomlinson, J. M., Gotzner, N., & Bott, L. (2017). Intonation and Pragmatic Enrichment: How Intonation Constrains Ad Hoc Scalar Inferences. *Language and Speech*, *60*(2), 200–223. https://doi.org/10.1177/0023830917716101

Tower-Richardi, S. M., Brunyé, T. T., Gagnon, S. A., Mahoney, C. R., & Taylor, H. A. (2012). Abstract spatial concept priming dynamically influences real-world actions. *Frontiers in Psychology*, *3*(SEP), 1–12. https://doi.org/10.3389/fpsyg.2012.00361

Travers, E., Rolison, J. J., & Feeney, A. (2016). The time course of conflict on the Cognitive Reflection Test. *Cognition*, *150*, 109–118. https://doi.org/10.1016/j.cognition.2016.01.015

Viswanathan, N., & Kelty-Stephen, D. G. (2018). Comparing speech and nonspeech context effects across timescales in coarticulatory contexts. *Attention, Perception, and Psychophysics*, *80*(2), 316–324. https://doi.org/10.3758/s13414-017-1449-8

Vogel, D., Scherbaum, S., & Janczyk, M. (2018). Dissociating decision strategies in free-choice tasks – A mouse tracking analysis. *Acta Psychologica*, *190*(July), 65–71. https://doi.org/10.1016/j.actpsy.2018.06.012

Wang, Q., Taylor, H. A., & Brunyé, T. T. (2012). When going the right way is hard to do: Distinct phases of action compatibility in spatial knowledge development. *Acta Psychologica*, *139*(3), 449–457. https://doi.org/10.1016/j.actpsy.2012.01.006

Wang, Q., Taylor, H. A., & Brunyé, T. T. (2020). Action compatibility in spatial knowledge developed through virtual navigation. *Psychological Research*, *84*(1), 177–191. https://doi.org/10.1007/s00426-018-0972-0

Ward, R. M., & Kelty-Stephen, D. G. (2018). Bringing the Nonlinearity of the Movement System to Gestural Theories of Language Use: Multifractal Structure of Spoken English Supports the Compensation for Coarticulation in Human Speech Perception. *Frontiers in Physiology*, *9*(SEP), 1–22. https://doi.org/10.3389/fphys.2018.01152

Weaver, S. M., & Arrington, C. M. (2013). Tracking the Multitasking Mind. *Zeitschrift Für Psychologie*, *221*(1), 51–60. https://doi.org/10.1027/2151-2604/a000130

Wifall, T., Buss, A. T., Farmer, T. A., Spencer, J. P., & Hazeltine, E. (2017). Reaching into response selection: Stimulus and response similarity influence central operations. *Journal of Experimental Psychology: Human Perception and Performance*, *43*(3), 555–568. https://doi.org/10.1037/xhp0000301

Xiao, K., & Yamauchi, T. (2014). Semantic priming revealed by mouse movement trajectories. *Consciousness and Cognition*, *27*(1), 42–52. https://doi.org/10.1016/j.concog.2014.04.004

Xiao, K., & Yamauchi, T. (2017). The role of attention in subliminal semantic processing: A mouse tracking study. *PLoS ONE*, *12*(6), 1–17. https://doi.org/10.1371/journal.pone.0178740

Yamamoto, N., Incera, S., & McLennan, C. T. (2016). A Reverse Stroop Task with Mouse Tracking. *Frontiers in Psychology*, *7*(MAY), 1–12. https://doi.org/10.3389/fpsyg.2016.00670

Yu, Z., Wang, F., Wang, D., & Bastin, M. (2012). Beyond reaction times: Incorporating mouse-tracking measures into the implicit asociation test to examine its underlying proces. *Social Cognition*, *30*(3), 289–306. https://doi.org/10.1521/soco.2012.30.3.289
